# LILRB2-mediated TREM2 signaling inhibition suppresses microglia functions

**DOI:** 10.1186/s13024-022-00550-y

**Published:** 2022-06-18

**Authors:** Peng Zhao, Yuanzhong Xu, Lu-Lin Jiang, Xuejun Fan, Zhiqiang Ku, Leike Li, Xiaoye Liu, Mi Deng, Hisashi Arase, Jay-Jiguang Zhu, Timothy Y. Huang, Yingjun Zhao, Chengcheng Zhang, Huaxi Xu, Qingchun Tong, Ningyan Zhang, Zhiqiang An

**Affiliations:** 1 Texas Therapeutics Institute, Brown Foundation Institute of Molecular Medicine, University of Texas Health Science Center at Houston, Houston, TX USA; 2 Center for Metabolic and Degenerative Diseases, Brown Foundation Institute of Molecular Medicine, University of Texas Health Science Center at Houston, Houston, TX USA; 3 Neuroscience Initiative, Sanford Burnham Prebys Medical Discovery Institute, La Jolla, CA 92037 USA; 4 Department of Physiology, UT Southwestern Medical Center, Dallas, TX USA; 5 Department of Immunochemistry, Research Institute for Microbial Diseases, Osaka University, Suita, Osaka Japan; 6 Department of Neurosurgery, University of Texas Health Science Center in Houston, McGovern Medical School and Memorial Hermann, Houston, TX USA; 7 State Key Laboratory of Cellular Stress Biology, Fujian Provincial Key Laboratory of Neurodegenerative Disease and Aging Research, Institute of Neuroscience, School of Medicine, Xiamen University, Xiamen, Fujian China

**Keywords:** Alzheimer’s disease, TREM2, ITAM, Antibody, LILRB2, ITIM, Phagocytosis, Microglia, Amyloid, 5XFAD mice

## Abstract

**Background:**

Microglia plays crucial roles in Alzheimer’s disease (AD) development. Triggering receptor expressed on myeloid cells 2 (TREM2) in association with DAP12 mediates signaling affecting microglia function. Here we study the negative regulation of TREM2 functions by leukocyte immunoglobulin-like receptor subfamily B member 2 (LILRB2), an inhibitory receptor bearing ITIM motifs.

**Methods:**

To specifically interrogate LILRB2-ligand (oAβ and PS) interactions and microglia functions, we generated potent antagonistic LILRB2 antibodies with sub-nanomolar level activities. The biological effects of LILRB2 antagonist antibody (Ab29) were studied in human induced pluripotent stem cell (iPSC)–derived microglia (hMGLs) for migration, oAβ phagocytosis, and upregulation of inflammatory cytokines. Effects of the LILRB2 antagonist antibody on microglial responses to amyloid plaques were further studied in vivo using stereotaxic grafted microglia in 5XFAD mice.

**Results:**

We confirmed the expression of both LILRB2 and TREM2 in human brain microglia using immunofluorescence. Upon co-ligation of the LILRB2 and TREM2 by shared ligands oAβ or PS, TREM2 signaling was significantly inhibited. We identified a monoclonal antibody (Ab29) that blocks LILRB2/ligand interactions and prevents TREM2 signaling inhibition mediated by LILRB2. Further, Ab29 enhanced microglia phagocytosis, TREM2 signaling, migration, and cytokine responses to the oAβ-lipoprotein complex in hMGL and microglia cell line HMC3. In vivo studies showed significantly enhanced clustering of microglia around plaques with a prominent increase in microglial amyloid plaque phagocytosis when 5XFAD mice were treated with Ab29.

**Conclusions:**

This study revealed for the first time the molecular mechanisms of LILRB2-mediated inhibition of TREM2 signaling in microglia and demonstrated a novel approach of enhancing TREM2-mediated microglia functions by blocking LILRB2-ligand interactions. Translationally, a LILRB2 antagonist antibody completely rescued the inhibition of TREM2 signaling by LILRB2, suggesting a novel therapeutic strategy for improving microglial functions.

**Supplementary Information:**

The online version contains supplementary material available at 10.1186/s13024-022-00550-y.

## Background

Alzheimer’s disease (AD), the main cause of dementia, is a neurodegenerative disorder with limited treatment options [1, 2]. Key pathological features of AD include progressive memory loss, cognitive deficits, synapse loss [3], neuronal death [4], β-amyloid (Aβ) plaque deposition [5], and hyperphosphorylated tau protein forming neurofibrillary tangles [6, 7]. Microglia, brain-resident myeloid cells, play pivotal roles in AD development by regulating brain homeostasis [8] and neuroinflammation [9], promoting Aβ plaque phagocytic clearance [10], and clustering and limiting diffusion of Aβ plaques [11] and tau tangles [12]. Microglia genetic variants, including genes CR1, CD33, and TREM2, contribute to the pathogenesis of late-onset AD (LOAD) [13]. Among the microglia genes, polymorphisms of TREM2, a transmembrane protein selectively expressed in microglia, have been associated with an increased risk of developing AD [14].

TREM2 affects AD by its regulation of microglia functions. TREM2, a single-pass transmembrane receptor expressed in microglia, binds to a variety of ligands, e.g., lipoproteins, phospholipids, and oligomeric Aβ (oAβ) [14]. The transmembrane region of TREM2 is associated with adaptor protein DNAX-activation protein 12 (DAP12) [14]. Upon cross-linking by ligands, phosphorylation of immunoreceptor tyrosine-based activation motifs (ITAM) within DAP12 recruits and activates kinases. Further, this phosphorylation induces cell activation by initiating signal cascades involving Ca^2+^ mobilization, cytoskeleton rearrangement, MAPK signaling, and activation of energetic metabolism. TREM2 signaling significantly affects microglia cellular phenotypes and functions, including phagocytosis, lipid metabolism, metabolic shift [14]. As a result, TREM2 promotes cell survival and counteracting inflammatory activation.

Leukocyte immunoglobulin-like receptor subfamily B member 2 (LILRB2), an inhibitory receptor bearing three immunoreceptor tyrosine-based inhibitory motifs (ITIM), was found to be expressed selectively in monocytes, macrophages, and dendritic cells [15]. LILRB2 transduces negative signals by its association with SHP-1 phosphatase via the ITIM motifs [15]. LILRB2 has been shown to play important roles in neurological function [16]. PirB, LILRB2 ortholog in mice, is expressed on neuronal growth cones and is associated with synapses [16]. Both human LILRB2 and mouse PirB bear ITIM motifs that can attenuate signaling cascades receptors bearing ITAM when the two receptors are cross-linked [17, 18]. oAβ contributed to memory deficits in adult mice and mediates synaptic plasticity loss in the juvenile visual cortex through a PirB-dependent manner in transgenic mice [16]. Smith and colleagues showed that LILRB2 was one of the three major receptors showing strong interaction with Aβ oligomers [19, 20]. Kim and colleagues found that oAβ binds to both LILRB2 and PirB with nanomolar affinity [16]. Ligands shared between ITIM and ITAM receptors can induce inhibition to the ITAM receptor upon clustered with the ITIM receptor [17, 18]. LILRB2 was shown to deliver inhibitory functions when co-engaged with FcγRII with a significant reduction of Ca^2+^ mobilization triggered via FcγRII [15]. LILRB2 was also shown to inhibit macrophage functions including phagocytosis [21]. Chen and colleagues showed that LILRB2 antagonism inhibited SHP1/2 recruitment and generated inflammatory macrophages in the presence of M-CSF [21].

In this study, we report the co-expression of LILRB2 and TREM2 in human microglia. Both microglia from AD patients and normal subjects express LILRB2 and TREM2. Ligands shared between LILRB2 and TREM2 were found to induce TREM2 and LILRB2 co-ligation and induced signal inhibition mediated by LILRB2, negatively affecting microglia functions. A LILRB2 blocking monoclonal antibody was shown to completely prevent LILRB2-mediated TREM2 signaling inhibition by blocking ligand-LILRB2 interactions and promoting microglia functions in vitro and in vivo. Taken together, these results suggest that the LILRB2 blocking antibody has the potential for AD treatment by improving TREM2-mediated microglial functions.

## Methods

### Cell lines

HEK293T and HMC-3 cell lines were acquired from the American Type Culture Collection (ATCC) and cultured in DMEM+ 10% FBS. BV2 was cultured in DMEM+ 10% FBS. The 2B4 NFAT-GFP reporter cell line was cultured in RPMI-1640 + 10% FBS.

### Immunostaining of human brain tissue

Human brain tissues were purchased from Novusbio as prepared slides. For normal subject brain tissues, catalog# NBP2-77565 was used. For AD patient brain tissues, catalog# NBP2-78018 was used. Before immunostaining, the slides were deparaffinized and rehydrated according to a previously published protocol [22]. Antigen retrieval was done using 88% formic acid following a previously published protocol [23].

After antigen retrieval, slides were first blocked in 1% BSA PBS with 0.3% Triton X-100 for 2 hours, then stained with corresponding antibodies: IBA1 (1:1000, Abcam, ab5076), 6E10 (1:500, Biolegend), GFAP (1:1000, Thermo Fisher PA1-10004), LILRB2 (1:500, Thermo Fisher 16-5149-85) and TREM2 (1:1000, Thermo Fisher, 27,599-1-AP) in 1% BSA PBS with 0.3% Triton X-100 overnight at 4 °C in a humidity chamber. After washing in PBS 0.3% Triton X-100, corresponding secondary antibodies with fluorescent labeling were incubated with brain slices for 2 hours at 4 °C in a humidity chamber. The slides were then mounted using ProLong Gold Antifade Mountant (Thermo Scientific). The slides were imaged using a Leica TCS SP5 confocal microscope.

### Fc fusion proteins and oAβ

LILRB2- and TREM2-Fc fusion proteins were constructed by fusing the extracellular domain (ECD) of the receptors to human IgG1 Fc. The fusion protein gene was cloned into a vector that drives protein expression by a CMV promoter. Proteins were expressed in Expi 293 cells and purified by protein A agarose to purity > 95% confirmed by SDS-PAGE.

Oligomer Aβ (oAβ) was prepared according to protocols previously reported [24, 25]. Briefly, unlabeled β-amyloid (1-42), 5-FAM-labeled β-amyloid (1-42), or biotin-β-amyloid (1-42) were purchased from AnaSpec as a lyophilized powder. The lyophilized powder was dissolved in HFIP (1,1,1,3,3,3-Hexafluoro-2-propanol) and then dried under a vacuum to dissolve pre-existing aggregates. The dried β-amyloid peptide was then reconstituted in DMSO to prepare β-amyloid monomers and stored at − 80 °C until use. To prepare oligomer β-amyloid, the DMSO-dissolved β-amyloid monomers were diluted to DPBS and incubated at 37 °C overnight.

### Ligands binding to LILRB2 and TREM2 as measured by ELISA

For oAβ ELISA, 2 μg/mL protein A (Sino Biological) was coated on a high-binding ELISA plate by incubation overnight at 4 °C. After blocking with 1% BSA PBS, LILRB2- or TREM2-Fc fusion protein was captured by incubation at 2 μg/mL for 1 hour at room temperature. After washing with PBS + 0.05% Tween-20, biotinylated oAβ was added at designated concentrations and incubated for 1 hour at room temperature. After washing with PBS + 0.05% Tween-20, streptavidin-HRP (R&D Systems) was added at 1:200 concentration and incubated for 1 hour at room temperature. After washing with PBS + 0.05% Tween-20, TMB substrate (Thermo Fisher Scientific) was added and incubated for 5 min before the reaction was stopped by the addition of 1 N H_2_SO_4_. Optical density (OD) values were read at 450 nm.

For the PS (L-α-phosphatidylserine, purchased from Avanti Polar Lipids) ELISA, 0.1 mg/mL of PS dissolved in methanol was coated onto a high-binding ELISA plate by evaporating at room temperature for 4 hours. After blocking with 1% BSA PBS, LILRB2- or TREM2-Fc fusion protein was allowed to bind by incubating at designated concentrations for 1 hour at room temperature. After washing with PBS + 0.05% Tween-20, anti-human Fc-HRP (Jackson ImmunoResearch) was added at 1:10,000 dilution and incubated for 1 hour at room temperature. After washing with PBS + 0.05% Tween-20, TMB substrate (Thermo Fisher Scientific) was added and incubated for 5 min before the reaction was stopped by the addition of 1 N H_2_SO_4_. OD values were read at 450 nm.

### Ligands binding to LILRB2 and TREM2 as measured by BLI

LILRB2- or TREM2-Fc fusion proteins were diluted to 30 μg/mL in kinetics buffer. PS liposomes were prepared as previously reported with modifications [26]. Briefly, PS was dissolved in chloroform and dried under a vacuum to form a thin layer. DPBS was added to re-hydrate PS, and the liposomes were formed by sonication on ice until the solution became translucent. Octet Red 96 instrument (Fortebio) was used in the bio-layer interferometry (BLI) assay. Protein A sensors (Fortebio) were used to capture Fc fusion proteins. During all incubation steps, samples were kept at room temperature with 1000 rpm shaking.

In the association stage of the BLI assay, protein A sensor-captured Fc fusion proteins were incubated with oAβ (1 μM in kinetics buffer) or PS liposome (1 mM in kinetics buffer) for the designated time. After the association stage, the sensors were dipped into a ligand-free kinetics buffer to allow the bound ligand oAβ or PS to freely dissociate for the designated time.

For measuring the kinetics of LILRB2 or TREM2 binding with oAβ, protein A sensor-captured Fc fusion proteins were incubated with oAβ at multiple concentrations for the designated time. After the association stage, the sensors were dipped into a kinetics buffer to allow the bound ligand oAβ to freely dissociate for the designated time. The binding kinetics parameters were calculated by analysis software from Fortebio (version 11) using a 1:1 binding model. KD was calculated as k_dis_/k_on_.

### Panning of phage-displayed scFv libraries for LILRB2 specific antibodies

A phage-displayed scFv antibody library was prepared previously [27]. Panning of the library for LILRB2 specific antibodies was carried out as described previously with modifications [27]. Briefly, MaxiSorp Nunc-Immuno tubes (Thermo Fisher Scientific) were coated with 20 μg/mL LILRB2-Fc in DPBS overnight at 4 °C. Unbound antigen was removed after washing with DPBS. After blocking the surface with 5% milk in DPBS, the phage library was incubated with the coated-LILRB2 for 2 hours at room temperature in 5% milk with 0.1 mg/mL D3-D4-Fc. After washing with PBS + 0.05% tween-20 to remove unbound phage, captured phage was eluted by incubating with 100 mM TEA for 20 min. Eluted phage-infected log-phase growing *E. coli* TG1 and then were amplified on 2x YTAG agar 500cm^2^ square plate (Corning) at 30 °C overnight. The amplified phage-infected TG1 was used to prepare the phage for the next round of panning using the M13KO7 helper phage. The enrichment process was done in three rounds using the output from the previous round as the input for the next round.

After three rounds of panning, the output titer was measured and single colonies were used to prepare phage for ELISA. High-binding ELISA plates (Corning) were coated with LILRB2-Fc at 2 μg/mL overnight at 4 °C. After blocking with 5% milk in PBS, phage prepared from single TG1 colonies in 5% milk PBS was incubated with coated LILRB2 for 1 hour at room temperature. After washing with PBS + 0.05% Tween-20, anti-M13-HRP (Santa Cruz Biotechnology) was added at 1:2000 concentration and incubated for 1 hour at room temperature. After washing with PBS + 0.05% Tween-20, TMB substrate (Thermo Fisher Scientific) was added and incubated for 5 min before being stopped by 1 N H_2_SO_4_. OD values were read at 450 nm. Top 20% high-binding clones were selected. Phagemids were extracted using Qiagen BioRobot Universal System in 96-well format. After DNA sequencing, sequences were analyzed using the IMGT V-quest service to identify antibody sequences with unique CDR3 regions.

### Conversion of phage scFv to IgG

Unique scFv clones were converted into human IgG1 using mixed universal primers with degeneracy [27]. Individual heavy and light variable chains were amplified using PrimeStar GXL polymerase (Takara Bio). Gel-purified variable chain fragments were cloned into digested vectors using In-fusion HD cloning enzyme mix (Takara Bio). After the converted plasmid was sequenced, sequences verified IgG plasmids were transfected into Expi 293 cells at the 2-mL scale. After culturing for 5 days, cells were removed and antibody-containing supernatant was collected for screening assay.

For milligram-scale antibody purification, Expi293-produced antibodies were purified using CaptivA Protein A affinity resin (Repligen) and eluted with 0.1 M glycine (pH = 2.5) and then neutralized with 1/20 volume 1 M Tris-HCl (pH = 9). Buffer exchange to DPBS was done using Amicon Ultra-15 ultrafiltration units (Mw cutoff = 30 k) (MilliporeSigma).

### NFAT-GFP reporter assay

The chimeric LILRB2 reporter construct was generated by fusing LILRB2 (aa22-461) with helical and cytoplasmic regions of human TREM2 (aa175-230). The original signal peptide of LILRB2 was replaced by leader sequence from mouse immunoglobulin κ light chain. A HA tag was introduced to the N′ of LILRB2. For mutant LILRB2 reporter constructs, LILRB2 with designated mutations were prepared using In-fusion HD cloning enzyme mix with the WT LILRB2 reporter construct as the template.

The human TREM2-DAP12 reporter construct was generated by fusing human TREM2 (aa19-169) with huDAP12 (aa28-113) with D50A mutation. The original signal peptide of TREM2 was replaced by leader sequence from mouse immunoglobulin κ light chain. A HA tag was introduced to the N-terminus of TREM2. The mouse TREM2-DAP12 reporter construct was generated in a similar design except for mouse TREM2 (aa19-171) replaced the human TREM2.

The co-ligation reporter cell lines LILRB2-TREM2 or LILRB2_muITIM-TREM2 were generated by transducing co-ligation reporter constructs into the parental 2B4 NFAT-GFP reporter cells. The co-ligation reporter construct enabling WT LILRB2 and TREM2 co-expression was generated by fusing WT LILRB2 (aa1-598), IRES, and human TREM2-DAP12 (Igk-HA-huTREM2 (19-169)-huDAP12 (aa28-113, D50A)). IRES enables co-expression of both receptors (LILRB2 and TREM2) in the same reporter cell. Similarly, the co-ligation reporter construct enabling LILRB2_muITIM and TREM2 co-expression was generated by fusing LILRB2_muITIM (LILRB2 with mutations Y533F, Y562F, and Y592F), IRES, and TREM2-DAP12 (Igk-HA-huTREM2 (aa19-169)-huDAP12 (aa28-113, D50A)).

All chimeric reporter genes were cloned into pCDH-CMV-MCS-EF1α-Puro. The 2B4 reporter cells transduced with individual reporter constructs were generated by lentivirus transduction. To prepare lentivirus particles, pCMV-VSV-G (Addgene 8454), pCMV delta R8.2 (Addgene 12,263), and individual pCDH transfer plasmids containing GOI were transfected into HEK293T. The 2B4 NFAT-GFP parental reporter cells were transduced with lentivirus supernatant (1:1 diluted in RPMI-1640) overnight under the presence of 10 μg/mL polybrene (Santa Cruz Biotechnology). After 48 hours of transduction, cells were selected with 1 μg/mL puromycin until a sufficient number of cells with transgene emerged.

For the reporter assay, ligands were coated onto 96-well cell culture plates at their optimal concentrations determined in preliminary experiments: oAβ (1 μM in DPBS, overnight, 4 °C), PS (0.1 mg/mL in methanol, room temperature until fully evaporated), PC (L-α-phosphatidylcholine, purchased from Avanti Polar Lipids, 0.03 mg/mL in methanol, room temperature until fully evaporated), and antibodies (10 μg/mL, DPBS, overnight, 4 °C). After ligand coating, unbound ligands were removed by washing with DPBS 3 times. A total of 100,000 reporter cells were seeded into individual wells (96-well plate) in 0.1 mL complete medium with 1 μg/mL puromycin with designated treatments. After overnight culturing, GFP positive populations were read using an iQue3 high throughput flow cytometer (Sartorius) with at least 10,000 live cells collected.

### Blocking of ligand-receptor interaction by LILRB2 antibodies as measured by BLI

Protein A sensors (Fortebio) were used to capture Fc fusion proteins. During all incubation steps, samples were kept at room temperature with 1000 rpm shaking. For assays with oAβ as the ligand, individual purified LILRB2 antibodies were loaded onto sensors at 30 μg/mL. After antibody capture, unbound Protein A was blocked by incubating in 0.1 mg/mL human Fc fragment. The sensor-captured antibodies were then incubated with LILRB2-Fc at 200 nM in kinetics buffer for 120 seconds to load LILRB2. The LILRB2-loaded sensors were incubated with biotin-oAβ at 1 μM in kinetics buffer for the designated time. The sensors were incubated with 200 nM streptavidin to further amplify oAβ binding signals. For assays with PS as the ligand, the only difference is that PS liposomes (1 mM PS concentration) in kinetics buffer were incubated with LILRB2-loaded sensors instead of oAβ, and there was no streptavidin-based signal amplification step. After PS binding to LILRB2, the sensors were incubated in a blank kinetics buffer to allow free dissociation of PS.

### Antibody LILRB2 binding affinity measured by ELISA

High-binding ELISA plates (Corning) were coated with LILRB2-Fc at 2 μg/mL overnight at 4 °C. After blocking with 1% BSA PBS, individual purified LILRB2 antibodies (at designated concentrations) in 1% BSA PBS were incubated with coated LILRB2 for 1 hour at room temperature. After washing with PBS + 0.05% Tween-20, anti-human F(ab)2-HRP (Jackson ImmunoResearch) was added at 1:5000 concentration and incubated for 1 hour at room temperature. After washing with PBS + 0.05% Tween-20, TMB substrate (Thermo Fisher Scientific) was added and incubated for 5 min before being stopped by 1 N H_2_SO_4_. OD values were read at 450 nm.

### Antibody LILRB2 binding kinetics measured by BLI

Protein A sensors (Fortebio) were used to capture individual Fc fusion proteins. During all incubation steps, sample temperature was set to room temperature with 1000 rpm shaking. Individual purified LILRB2 antibodies were loaded onto sensors at 30 μg/mL. After antibody capture, the antibody-loaded sensors were incubated with LILRB2-His at designated concentrations for the defined time. After LILRB2 binding, the sensors were incubated in a blank kinetics buffer to allow free dissociation of LILRB2 for the designated time. The binding kinetics parameters were calculated by the Fortebio software (version 11) using a 1:1 binding model with global fitting. KD is calculated by k_dis_/k_on_.

### Antibody binding to LILRB2 expressed on the cell surface

To prepare LILRB2-expressing cells, HEK293T cells were transfected with pcDNA3.1 carrying LILRB2 (aa1-598) with CMV-driven expression. After transfection for 72 hours, purified LILRB2 antibody (at designated concentration) in 1% BSA DPBS was incubated with transfected cells for 30 min on ice. Unbound antibody was removed by centrifuging cells at 350 rpm for 5 min. Then, Alexa Fluor 488-anti-human IgG (H + L) in 1% BSA DPBS (Thermo Fisher Scientific) at 2 μg/mL was incubated for 30 min on ice. After centrifuging cells at 350 rpm for 5 min to remove unbound secondary antibody, cells were analyzed using the iQue3 high throughput flow cytometer (Sartorius) with at least 10,000 live cells collected. The control cell line was transfected with a blank pcDNA3.1 plasmid.

### Antibody LILRB2 specificity measured by BLI

We measured the cross-reactivity of LILRB2 antibodies to members of the LILRB and LILRA family of proteins by the BLI assay. Protein A sensors (Fortebio) were used to capture individual LILRB2 antibodies. During all incubation steps, sample temperature was set to room temperature with 1000 rpm shaking. Individual purified LILRB2 antibodies were loaded onto sensors at 30 μg/mL. After antibody capture, unbound protein A was blocked by incubating in 0.1 mg/mL human Fc fragment. Then the antibody-loaded sensors were incubated with purified Fc fusion proteins of ECD LILRB and LILRA receptor family members at 200 nM in kinetics buffer for the defined time. After LILRB2 binding, the sensors were incubated in a blank kinetics buffer to allow free dissociation of Fc fusion proteins for the designated time.

### Antibody LILRB2 specificity measured by ELISA

High-binding ELISA plates (Corning) were coated with purified ECD-Fc fusion proteins of LILRB and LILRA receptor family members at 2 μg/mL overnight at 4 °C. After blocking with 1% BSA PBS, individual purified LILRB2 antibodies at 4 nM in 1% BSA PBS were incubated with coated antigens for 1 hour at room temperature. After washing with PBS + 0.05% Tween-20, anti-human F(ab)2-HRP (Jackson ImmunoResearch) was added at 1:5000 concentration and incubated for 1 hour at room temperature. After washing with PBS + 0.05% Tween-20, TMB substrate (Thermo Fisher Scientific) was added and incubated for 5 min before being stopped by 1 N H_2_SO_4_. OD values were read at 450 nm.

### Preparation of oAβ-lipoprotein complexes

L-α-phosphatidylserine (PS) and 1,2-dimyristoyl-sn-glycero-3-phosphocholine (DMPC) were purchased from Avanti Polar Lipids as powder. PS and DMPC were dissolved in chloroform at 10 mg/mL and mixed at 1:4. Chloroform was evaporated under vacuum and formed a thin layer containing the mixture of PS and DMPC. DPBS was added to re-hydrate the lipid mixture to 5 mg/mL, and the liposomes were formed by sonication on ice until the solution becomes translucent. To prepare oAβ-lipoprotein complexes, PS/DMPC liposomes and APOE3 were mixed at final concentrations of 1 mg/mL for PS/DMPC liposomes and 0.25 mg/mL for APOE3. The mixture was incubated at 18 °C 15 min and 30 °C 15 min for 3 cycles [28]. Then FAM-labeled oAβ was added into the lapidated APOE at a final concentration of 1 μM and incubated at room temperature for 1 hr.

### Immunoblot assays

HMC3 cells were seeded into a 6-well plate at 1 × 10^6^ cells/well in EMEM without FBS. After 1-hour incubation with designated treatments, the supernatant was removed, and cells were washed three times by DPBS. The cell lysate was obtained by lysing cells using NP-40 lysis buffer (1% NP40, 50 mM Tris-HCl, pH = 8, 150 mM NaCl) with Halt™ Protease and Phosphatase Inhibitor Cocktail (100X) (ThermoFisher). After removing debris by centrifugation, the total protein amount normalized by Pierce BCA Protein Assay Kit (ThermoFisher). Protein samples were resolved by 10% SDS-polyacrylamide gels (Biorad) and later transferred onto Immun-Blot PVDF membranes (Biorad). Proteins were probed with specific primary antibodies and secondary antibodies diluted in 5% BSA TBST [21, 24, 29]. The primary antibodies used are: SYK (1:1000, Cell Signaling 13198S), Phospho-Syk (Tyr525/526) (1:1000, Thermo Fisher MA5-14918), β-Actin (1:1000, Cell Signaling 4970S), SHP1 (1:1000, Cell Signaling 3759S), Phospho-SHP-1 (Tyr564) (1:1000, Cell Signaling 8849S), and LILRB2 (1:1000, Thermo Fisher PA5-46983).

The immunoreactive bands were visualized with the West Pico PLUS Chemiluminescent Substrate (ThermoFisher). The immunoreactive bands were quantified using ImageJ. Three independent treatment replicates were conducted with the representative immunoblot shown.

### Imaging of 293 T cells for oAβ and PS binding

LILRB2 or TREM2 expressing HEK293T cells were seeded into 8-well chamber slides (Lab-Tek) at 20,000 cells/well in DMEM+ 10% FBS overnight. The cells were then washed with DPBS and fixed with 4% PFA for 10 min at room temperature. For oAβ binding, Hilyte Fluor 488 labeled oAβ (prepared as described above using monomer Anaspec AS-60479-01) was added to 0.2 μM. For PS binding, PS liposomes (prepared as described above) were added to 1 μM. During incubation, 1% BSA PBS was used as the diluent to avoid non-specific binding. After incubation for 1 hour at room temperature, the solution containing ligands was removed, and cells were then washed twice with DPBS. For detecting the PS binding, FITC Annexin V (1:20, Biolegend) was incubated with cells for 30 min before nucleus staining. The nucleus was stained with TO-PRO-3 (1 μM) in DPBS for 30 min and then mounted using ProLong Gold Antifade Mountant (Thermo Scientific). Slides were imaged using a Leica TCS SP5 confocal microscope.

### Microglia phagocytosis assay

A total of 10,000 cells/well (BV2 with transgene or HMC3) were seeded in 96-well plates in serum-free DMEM medium overnight. oAβ-lipoprotein complex was diluted in serum-free DMEM medium with 1% BSA to a concentration equivalent to 100 nM FAM-oAβ. The medium in the cell culture plate was replaced with the diluted oAβ-lipoprotein complex and incubated at 37 °C for 2 hours. After phagocytosis, cells were detached by trypsin for 5 min, and cell surface-bound FAM-oAβ was quenched by adding trypan blue to 0.2% and incubated for 5 min. Cells were then transferred into a V-bottom 96-well plate and washed twice by 350 g 5 min centrifugation. When antibody treatment is needed, 10 μg/mL final antibody concentration was diluted together with the oAβ-lipoprotein complex. With CytoD treatment, serum-free DMEM medium (with 1% BSA) containing 10 μM CytoD was pre-incubated with cells for 30 min at 37 °C. CytoD was also included together with the oAβ-lipoprotein complex during phagocytosis. The phagocytosis was quantified using the iQue3 high throughput flow cytometer (Sartorius) with at least 10,000 live cells collected. Anti-LILRB2 42D1 was purchased from Thermo Fisher with catalog number 16-5149-85. The TREM2 blocking antibody was purchased from Millipore Sigma with catalog number MABN755.

For phagocytosis experiments using hMGL, cryopreserved iCell Microglia (R1131, FujiFilm) [30, 31] were seeded in a poly-D-lysine coated 96-well plate at 10,000 cells/well in basal medium supplied in the kit overnight. oAβ-lipoprotein complex was diluted to a concentration equivalent to 100 nM FAM-oAβ with 1% BSA. The medium in the cell culture plate was replaced with the diluted oAβ-lipoprotein complex and incubated at 37 °C for 2 hours. After phagocytosis, cells were detached by trypsin for 5 min, and cell surface-bound FAM-oAβ was quenched by adding trypan blue to 0.2% and incubated for 5 min. Cells were then transferred into a V-bottom 96-well plate and washed twice by 350 g 5 min centrifugation. With CytoD treatment, 10 μM CytoD was pre-incubated with cells for 30 min at 37 °C and constantly present during the phagocytosis experiment. The phagocytosis was quantified using an iQue3 high throughput flow cytometer (Sartorius).

### BV2 and HMC3 cell surface LILRB2 and TREM2 expression

BV2-LILRB2 or HMC3 cells were detached from culture plates by non-enzymatic dissociation buffer (Thermo Fisher Scientific). A total of 1 million LILRB2-BV2 or HMC3 cells were blocked by 0.1 mg/mL human Fc fragment in 1% BSA PBS for 30 min on ice to block Fc receptors. Then, primary antibodies (biotinylated anti-LILRB2, anti-TREM2, or control IgG) were added at a final concentration of 10 μg/mL and incubated for 30 min on ice. After centrifugation to remove unbound primary antibodies, Alexa Fluor 488-labeled streptavidin (Jackson ImmunoResearch) was added to 2 μg/mL in 1% BSA PBS for 30 min on ice. After removal of unbound streptavidin by centrifugation, cells were analyzed using an iQue3 high throughput flow cytometer (Sartorius) with at least 10,000 live cells collected.

### HMGL migration in transwell assays

Cryopreserved iCell Microglia (R1131, FujiFilm) [30, 31] were seeded in a transwell insert (PET membrane, 8 μm pore size, Corning 3374) in basal medium supplied in the kit without cytokines. Corresponding treatments were added into both the migration and receiver chambers at designated concentrations. Only the receiver (bottom) chambers contain a 0.5 μM oAβ-lipid complex. Cells were cultured for 24 hours at 37 °C with 5% CO_2_. After incubation, cells were washed three times with DPBS, fixed in 4% PFA for 10 min, and then stained with 0.05% crystal violet for 10 min. Unbound crystal violet was removed by washing with DPBS, and the plate was allowed to air-dry. Cell number was quantified by eluting cell-bound crystal violet in 33% acetic acid in H_2_O (100 rpm shaking, 10 min) according to the manufacturer’s protocol and literature [32]. The amount of crystal violet was quantified by measuring absorbance at 590 nm using a plate reader. For quantifying migrated cells, unmigrated cells that remain inside the transwell insert were removed using moistened cotton swabs. Migration percentage was calculated by dividing OD values of migrated cells over OD values of total cells.

For imaging microglia migration, the assay was conducted similarly as mentioned above, except that the microglia cells were pre-labeled with 1 μM CFSE (Thermo) for 15 min at 37 °C. The migrated cells were imaged using Nikon Eclipse TE2000E Widefield Fluorescence Microscope.

### Proximity ligation assays (PLA)

The PLA was performed according to the manufacturer’s protocol. HMGLs were seeded into 8-well chamber slides (Lab-Tek) in basal medium (as described in the method section “microglia phagocytosis assay”) overnight. Ligand oAβ-lipoprotein complex was diluted to a concentration equivalent to 100 nM FAM-oAβ with 1% BSA. The medium in the cell culture plate was replaced with the diluted oAβ-lipoprotein complex and incubated at 37 °C for 2 hours. If antibody treatment is applied, Ctrl IgG or Ab29 at 10 μg/mL final antibody concentration was diluted together with the oAβ-lipoprotein complex. After incubation, cells were fixed with 4% PFA for 10 min at room temperature and then permeabilized with 0.1% saponin for 10 min. Between all the incubation steps, cells were washed in 1% BSA in PBS with 0.05% Tween-20. PLA kit Duolink In Situ Red Starter Kit Mouse/Rabbit (DUO92101-1KT, Sigma) was applied to detect the clustering of LILRB2 and TREM2 according to the manufacturer’s protocol. Detection antibodies for LILRB2 and TREM2 were 42D1 (sc-53,594, rat isotype, compatible with the PLA kit, SCBT) and PA5-87933 (Thermo Fisher, rabbit isotype, compatible with the PLA kit), respectively. Saponin at 0.1% was always included in all the reagents and buffers during the PLA process. The LILRB2 and TREM2 antibodies were also pre-validated for not competing with ligands (oAβ and PS) or antibodies (Ctrl IgG and Ab29). The nucleus was stained with TO-PRO-3 (1 μM) in DPBS for 30 min and then mounted using ProLong Gold Antifade Mountant (Thermo Scientific). Slides were imaged using a Leica TCS SP5 confocal microscope.

### Real-time quantitative PCR analysis for cytokines in hMGL

Cryopreserved iCell Microglia (R1131, FujiFilm) [30, 31] were seeded in a poly-D-lysine coated 24-well plate in basal medium supplied in the kit without cytokines. hMGLs were incubated with the designated treatments for 24 hours before being collected for qPCR studies. RNA was extracted by RNeasy Micro Kit (Qiagen) following the manufacturer’s protocol. RNA was reverse transcribed into the first-strand cDNA using iScript™ cDNA Synthesis Kit (Biorad) following the manufacturer’s protocol. Quantitative PCR was performed using SsoAdvanced Universal SYBR Green Supermix (Biorad). Quantitative PCR reactions were measured by ABI PRISM 7900HT Sequence Detection System (ThermoFisher) using the standard protocol recommended by the manufacturer (95 °C, 30s, 40x cycles with 95 °C 15 s, 60 °C 30s per cycle with instrument default melting curve analysis). The following primers were used in the qPCR (primers are written in 5′ to 3′ directions): IL-1β-Forward: AGCTACGAATCTCCGACCAC, IL-1β-Reverse: CGTTATCCCATGTGTCGAAGAA; IL-6-Forward: ACTCACCTCTTCAGAACGAATTG, IL-6-Reverse: CCATCTTTGGAAGGTTCAGGTTG; TNF-α-Forward: CCTCTCTCTAATCAGCCCTCTG, TNF-α -Reverse: GAGGACCTGGGAGTAGATGAG; CCL3-Forward: AGTTCTCTGCATCACTTGCTG, CCL3-Reverse: CGGCTTCGCTTGGTTAGGAA. GAPDH was used as the internal control with the following primers: GAPDH-Forward: ACAACTTTGGTATCGTGGAAGG, GAPDH-Reverse: GCCATCACGCCACAGTTTC. Fold-of-change was calculated using the standard ΔΔCt method [24].

### 5XFAD mice studies

The animal experiments were conducted according to the institutional guidelines with approved protocols. 5XFAD mice (B6.Cg-Tg(APPSwFlLon,PSEN1*M146L*L286V)6799Vas/Mmjax, female, 8-week-old) were purchased from MMRRC and randomly grouped into 5 mice per group. Microglia implantation experiments started when the mice reached 5-mo-old age. To implant the microglia, 2 × 10^5^ hMGLs mixed with antibodies were injected into the bilateral ventricles under anesthesia. Stereotaxic injection of microglia (2 μL on each side) was delivered through a Neuro syringe (Hamilton 1701RN) into the lateral ventricles at a controlled speed of 0.5 μL/min. The antibody dose was 10 mg/kg per mouse. The mice were sacrificed 4 days after injection, and the brain tissues were harvested after mice transcardial perfusion at 2 mL/min by DPBS for 10 min. Brains were collected with half flash-frozen in liquid nitrogen and another half prepared for cryo-sectioning. For immunofluorescence, the half mouse brains were dipped into 4% PFA for 1d, then 30% sucrose for 2d before being embedded into OCT medium (Sakura) and sectioned using Leica Cryostat CM1950 into 40 μm floating coronal sections. The floating sections were stored at 4 °C in PBS with 0.01% sodium azide until use.

For immunostaining, after blocking in 1% BSA PBS with 0.3% Triton X-100 for 2 hours, brain sections were stained with corresponding antibodies: IBA1 (1:1000, Abcam, ab178680 [31, 33]), 6E10 (1:500, Biolegend), CD68 (1:500 Biolegend 333,812), human nuclear antigen (1:200, Abcam ab191181) and LILRB2 (1:1000, Thermo Fisher, PA5-97929) in 1% BSA PBS with 0.3% Triton X-100 overnight at 4 °C with gentle rocking. After washing in PBS 0.3% Triton X-100, corresponding secondary antibodies with fluorescent labeling were incubated with brain slices for 2 hours at 4 °C with gentle rocking. The nucleus was stained with TO-PRO-3 (1 μM) in DPBS for 30 min and then mounted using ProLong Gold Antifade Mountant (Thermo Scientific). Brains slices were imaged using a Leica TCS SP5 confocal microscope. The quantification was done using ImageJ as previously described [34, 35]. For quantification of the fluorescent intensity of indicated markers in the mouse cortex and hippocampus, images were analyzed by ImageJ, and background was subtracted by the software for fluorescence images before quantification. For quantification of Aβ engulfed by microglial CD68+ phagosome, the co-localization function of LAS X 3.7 (Leica) was used on Aβ+, CD68+ and Aβ + CD68+ image data. The ratio of engulfed Aβ by phagosome was calculated as the total Aβ + CD68+ area divided by the total Aβ + area. All the quantifications were done by an independent researcher blinded to the group designs.

For measuring the concentration of antibodies in the brain, high-binding ELISA plates (Corning) were coated with anti-human Fc (Jackson Immunoresearch) at 2 μg/mL overnight at 4 °C. After blocking with 1% BSA PBS, individual brain lysates were incubated with coated capture antigen for 2 hours at room temperature. After washing with PBS + 0.05% Tween-20, anti-human F(ab’)_2_-HRP (Jackson Immunoresearch) was added at 1:5000 concentration and incubated for 1 hour at room temperature. After washing with PBS + 0.05% Tween-20, TMB substrate (Thermo Fisher Scientific) was added and incubated for 5 min before being stopped by 1 N H_2_SO_4_. OD values were read at 450 nm. Standard curves were established for individual antibodies using purified corresponding antibodies following the same method listed above.

### Protein sequence analysis

Protein sequence alignment was performed using the T-Coffee multiple sequence alignment server and the alignment figures were generated in ESPript - http://espript.ibcp.fr [36]. The crystal structure was visualized using UCSF Chimera 1.15. The LILRB2 crystal structure was downloaded from the RCSB PDB database using entry 2gw5 [37].

### Statistical analysis

GraphPad Prism (v8, GraphPad Software) was used to generate plots and perform statistical analysis. Statistical differences were determined to be significant at *p* < 0.05 using a two-tailed Student t-test. Data are presented as mean ± SD.

## Results

### LILRB2 and TREM2 are expressed in human microglia

**Fig. 1:**
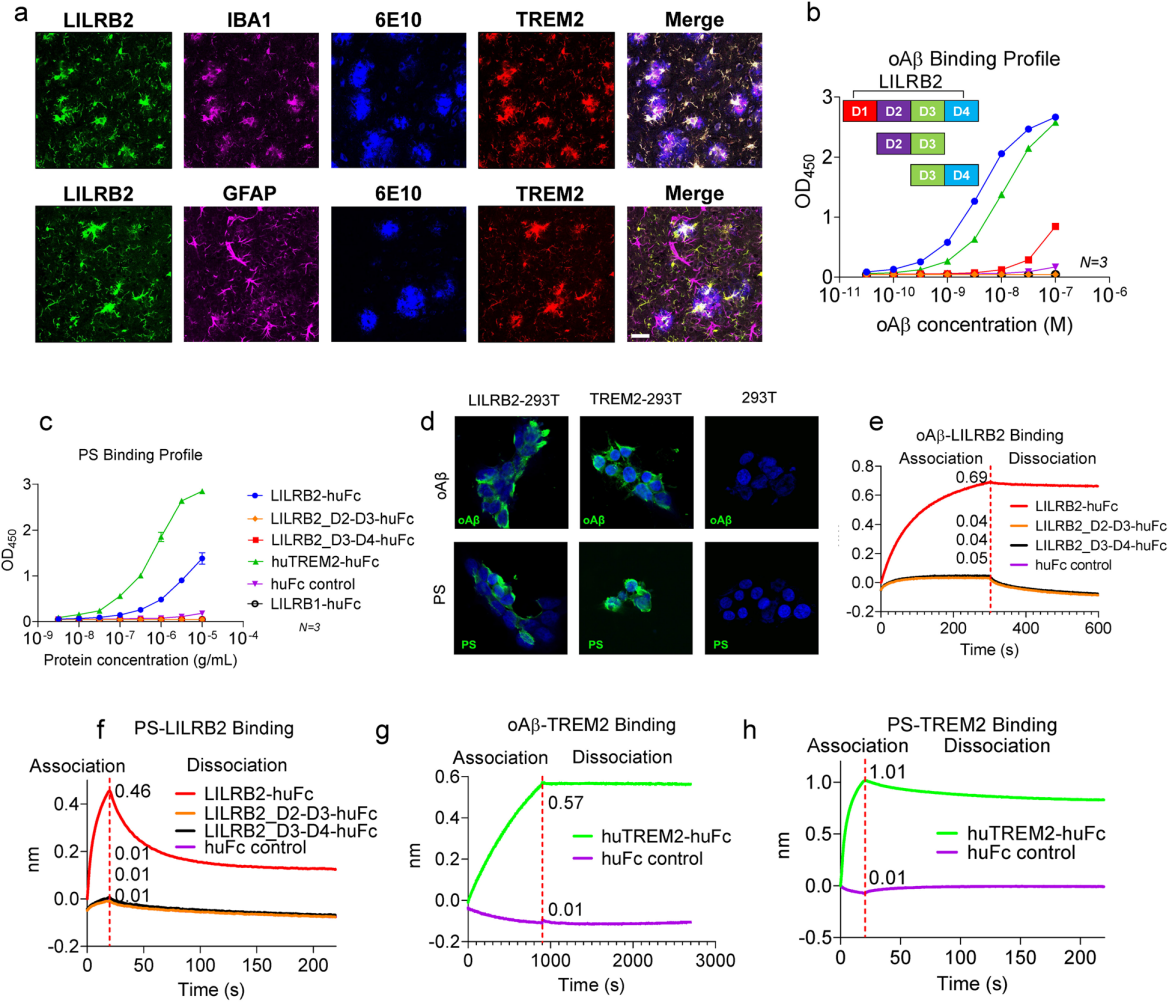
LILRB2 and TREM2 are expressed on human microglia and they share ligands oAβ and PS. **a**. Immunofluorescence staining of AD patient brain tissue for LILRB2, IBA1 (microglia marker), amyloid plaques (6E10), GFAP (astrocyte marker), and TREM2. Scale bar = 20 μm. **b**. Titration curves of oAβ binding to LILRB2 and TREM2 Fc fusion proteins by ELISA. Data are presented as mean ± SD (*n* = 3 independent experiments). **c**. Titration curves of PS binding to LILRB2 and TREM2 Fc fusion proteins by ELISA. Data are presented as mean ± SD (*n* = 3 independent experiments). **d**. Representative immunostaining images of oAβ and PS binding to LILRB2 and TREM2 expressed on 293 T cell surface. Scale bar represents 5 μm. **e**-**h**. oAβ or PS binding to LILRB2 or TREM2 as measured by BLI. In the association stage, protein A sensor-captured Fc fusion proteins (LILRB2-Fc: **e** and **f**, TREM2-Fc: **g** and **h**) were incubated with oAβ (1 μM, e and g) or PS liposomes (1 mM, **f** and **h**), and the amount of oAβ (**e** and **g**) or PS (**f** and **h**) bound onto the sensors was presented as wavelength shift in nanometers (nm). The red dotted vertical line marks the transit from association stage to dissociation stage, where the sensors were dipped into kinetics buffer without ligands allowing free dissociation

TREM2 has been reported previously to be expressed in microglia [38]. LILRB2 was known to be expressed by macrophages and monocytes [15], but there was no report on the status of LILRB2 expression in human microglia, which are myeloid cells inside the brain. To address this question, we used immunofluorescence staining to detect LILRB2 and TREM2 in the brain tissues of both AD patients and normal subjects. Results showed that microglia express both LILRB2 and TREM2 in brain tissue of AD patients (Fig. 1a) and normal subjects (Supplementary Fig. 1a). Both LILRB2 and TREM2 demonstrated co-localization with the microglia marker IBA1. As shown in Fig. 1a and Supplementary Fig. 1a, we stained AD patient and normal subject brain sections for LILRB2 and TREM2 together with the microglia marker IBA1. Both LILRB2 and TREM2 were found to co-localize with IBA1, indicating that human primary microglia express LILRB2 and TREM2, which is consistent with observation reported previously [38]. As controls, we observe no co-localization of either LILRB2 or TREM2 staining with the astrocytes marker GFAP (Fig. 1a and Supplementary Fig. 1a). More importantly, we observed the clustering of microglia around amyloid plaques (6E10-labeled) in the AD patient brain tissues (Fig. 1a).

### oAβ and PS bind to LILRB2 and TREM2

oAβ was shown previously to bind to both TREM2 and LILRB2 and exert biological functions [16, 24]. The recognition of a broad spectrum of lipid ligands by TREM2 was proven to be critical for microglia detection of damaged lipid patterns associated with neurodegeneration and to sustain microglia responses to Aβ accumulation [38]. TREM2 was shown to bind to PS, a phospholipid exposed to the outer leaflet of the plasma membrane during apoptosis [39–41]. We discovered that, in addition to oAβ, PS is also a ligand shared between LILRB2 and TREM2. ELISA results showed biotinylated oAβ binds strongly to surface captured LILRB2-Fc (EC_50_ = 3.81 nM, 95%CI 3.68 ~ 3.94 nM) and TREM2-Fc (EC_50_ = 10.94 nM, 95%CI 10.62-11.27 nM) (Fig. 1b). Similarly, plate-coated PS was able to bind to TREM2-Fc (EC_50_ = 0.68 μM, 95%CI 0.59-0.78 μM) (Fig. 1c). The binding affinity of plate-coated PS to LILRB2 was about 50% of that of TREM2-Fc, but PS still showed considerably higher binding to LILRB2 than to the huFc and LILIRB1 controls (Fig. 1c). In addition to ELISA, we validated the oAβ and PS interactions with LILRB2 and TREM2 in HEK293T overexpressing these two receptors. Using immunocytochemistry, both oAβ and PS showed significant binding to LILRB2 or TREM2-overexpressing HEK293T, but not to the parent HEK293T (Fig. 1d).

Using BLI, we further dissected the interaction kinetics between ligands and LILRB2 or TREM2. In comparison to the low binding signal of 0.04 for Fc control, the signal for oAβ binding to LILRB2 was 0.69 (Fig. 1e). Similarly, the signal for PS binding to LILRB2 was 0.46, which is much higher than the Fc control signal of 0.01 (Fig. 1f). oAβ and PS binding to TREM2 was also confirmed (Fig. 1g-h). PS used in the BLI assay was prepared as liposomes instead of plate-coated since liposomes closely resemble the natural state of PS in apoptotic cells or lipoprotein complex [42]. We next characterized the kinetics of oAβ binding to LILRB2 and TREM2. oAβ showed strong binding to both LILRB2 and TREM2 with binding affinity K_D_ = 69.3 nM and K_D_ = 12.6 nM for LILRB2 and TREM2, respectively (Supplementary Fig. 1b-c). Notably, oAβ showed almost no dissociation from LILRB2 and TREM2 (k_dis_ = 5.93 × 10^− 4^ 1/s for LILRB2 and k_dis_ = 1.23 × 10^− 4^ 1/s for TREM2, Supplementary Fig. 1b-c).

The LILRB2 ECD has four Ig-like domains, and we next mapped the functional domains responsible for the ligand binding. Identifying key domains allowed us to later focus the phage-displayed antibody library panning effort on the key domains and thus maximize the isolation of LILRB2 blocking antibodies. We expressed LILRB2 mutants with domain truncations and studied their binding to ligands in ELISA. As shown in Fig. 1b and c, deleting D1 and D2 (fusion protein with only D3 and D4) considerably reduced ligand-binding signals to a level similar to Fc control. Deleting D1 and D4 also abolished both the binding of LILRB2 to oAβ and PS (Fig. 1b-c). We next validated the LILRB2 domain mapping results using BLI. With a similar trend to the ELISA results, deleting D1 and D2 (fusion protein with only D3 and D4) showed considerably reduced signals (oAβ: 0.69 for LILRB2-Fc vs. 0.04 for D3-D4-Fc; PS: 0.46 for LILRB2-Fc vs. 0.01 for D3-D4-Fc) (Fig. 1e-f). Taken together, these results indicate that the D1 and D2 domains are critical to the binding between LILRB2 and its ligands (oAβ and PS).

### oAβ and PS induce LILRB2 and TREM2 co-ligation

**Fig. 2:**
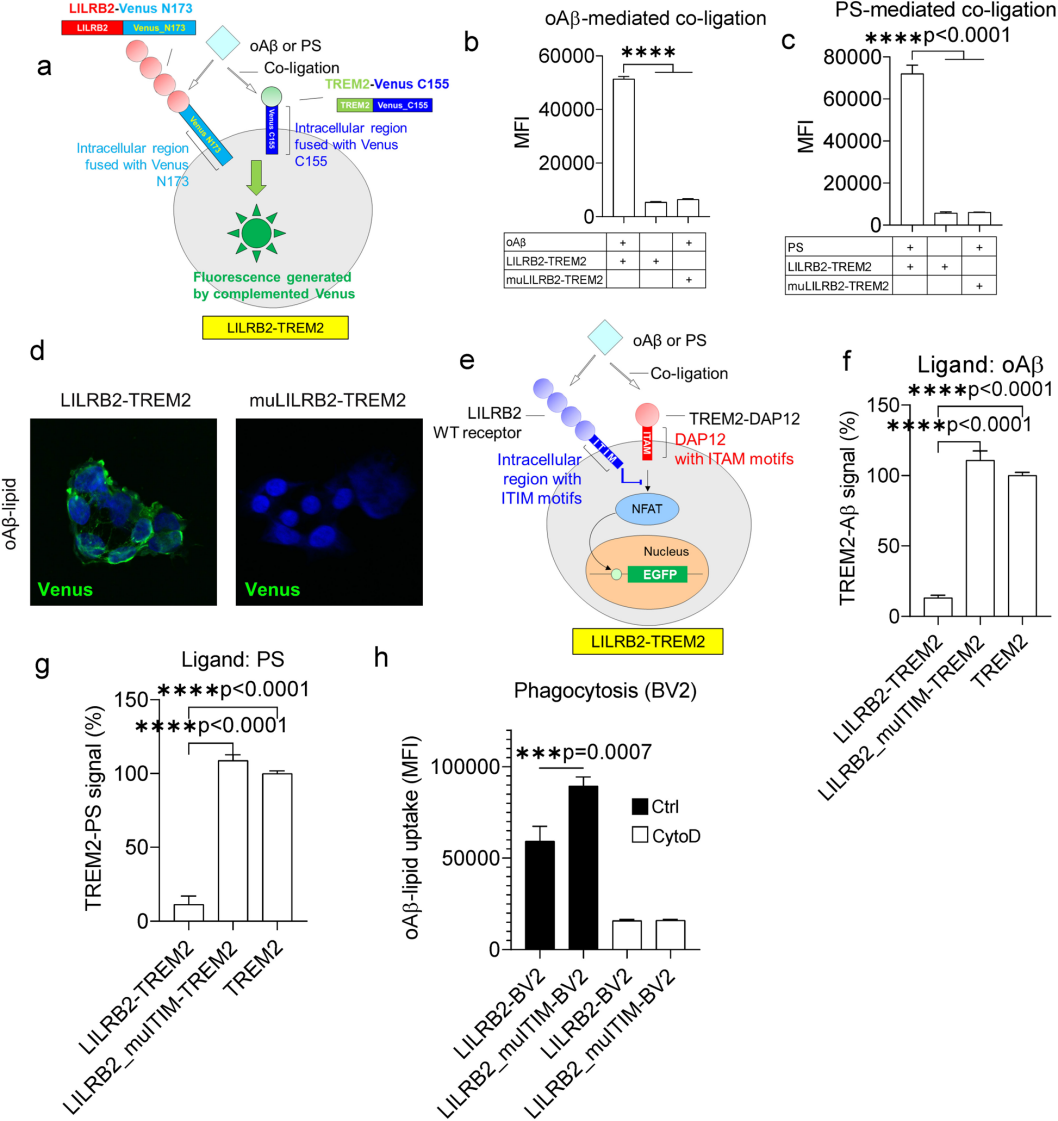
The shared ligands oAβ and PS induce co-ligation of LILRB2 and TREM2 and inhibit TREM2 signaling. **a**. Schematic illustration of the cell line LILRB2-TREM2 showing the design of BiFc assay with LILRB2 and TREM2 fusion constructs. **b**-**c**. oAβ or PS-induced co-ligation of TREM2 and LILRB2 as measured by the BiFc assay. HEK293T cells co-expressing LILRB2-N173 Venus and TREM2-C155 Venus were incubated with plate-coated oAβ (**b**) or PS (**c**). Y-axis shows the MFI signals from complemented Venus. Data are presented as mean ± SD (*n* = 3 independent experiments). **d**. Representative immunofluorescence images showing oAβ-lipid-induced co-ligation of TREM2 and LILRB2. Scale bar represents 5 μm. **e**. Schematic diagram showing co-ligation of LILRB2 and TREM2 by oAβ or PS inhibits TREM2 signaling in reporter cell assays. The ITIM motifs of LILRB2 attenuate signaling generated by ITAM motifs of TREM2 upon cross-linking by shared ligands oAβ or PS. **f**-**g**. oAβ or PS-induced TREM2 signaling of reporter cells co-expressing LILRB2 and TREM2. Reporter cells expressing corresponding receptors (x-axis) were incubated with oAβ (**f**) or PS (**g**). Y-axis is TREM2 signaling of treatment groups normalized based on the percentage of GFP^+^ reporter cells expressing only TREM2 (set to 100%). Data are presented as mean ± SD (*n* = 4 independent experiments). **h**. oAβ-lipoprotein complex phagocytosis profile of BV2 cells expressing the LILRB2 transgenes (x-axis). BV2 cells were incubated with FAM-oAβ-lipoprotein complex. Phagocytosis was quantified by flow cytometry, with the MFI shown. Negative phagocytosis control included 10 μM CytoD together with indicated treatments. Data are presented as mean ± SD (*n* = 4 independent experiments)

To validate that the shared ligands oAβ and PS induce co-ligation of LILRB2 and TREM2, we designed a co-ligation assay based on bimolecular fluorescence complementation (BiFc). In the BiFc assay, LILRB2 and TREM2 were fused with the N173 fragment of Venus and C144 fragment of Venus, respectively. The fusion gene was co-expressed in HEK293T cells. In the cell line named LILRB2-TREM2, WT LILRB2 was fused with the N173 fragment of Venus (Fig. 2a). In the cell line named muLILRB2-TREM2, a LILRB2 mutant with the D1 domain replaced by the D1 domain of LILRB1 was fused with N173 fragment of Venus (Supplementary Fig. 2a). The mutant was created to abolish ligand-LILRB2 interactions by replacing the LILRB2 D1 domain with the LILRB1 D1 domain, which does not bind to oAβ and PS, as demonstrated in Fig. 1b and c. Co-ligation of LILRB2 and TREM2 induced by oAβ, PS, or the oAβ-lipid exhibited a significant increase of fluorescence signal than that of when the ligand is absent (Fig. 2b-d). The co-ligation of LILRB2 and TREM2 is dependent on the binding between oAβ and LILRB2, as muLILRB2-TREM2 cells did not show a significant increase of fluorescent signals observed in the LILRB2-TREM2 cells upon receptor co-ligation (Fig. 2b-d).

### TREM2 signaling inhibition by shared ligand-induced co-ligation with LILRB2

To study the interactions between ligands and receptors in a cellular environment, we next studied LILRB2 and TREM2 activation by their shared ligands oAβ and PS using chimeric NFAT-GFP reporter cells. In the chimeric LILRB2 reporter construct, the ECD of LILRB2 was fused to the transmembrane and intracellular regions of human TREM2. Association with DAP12 allows ligands to trigger NFAT signaling and induce GFP expression by binding to extracellular LILRB2 [43, 44] (Supplementary Fig. 2b). oAβ significantly activated LILRB2 in the reporter cells as indicated by the high percentage of GFP^+^ cells over control (Supplementary Fig. 2c). PS also significantly activated LILRB2 in the reporter cells (Supplementary Fig. 2d). Notably, the response of LILRB2 to PC was similar to that of control reporter cells expressing an irrelevant chimeric receptor, which indicates that PC is not a ligand for LILRB2 (Supplementary Fig. 2d).

In the TREM2 NFAT-GFP reporter cells, the TREM2-DAP12 fusion gene was introduced, which delivers native TREM2 signaling through DAP12 (Supplementary Fig. 2e). The TREM2-DAP12 fusion was designed by fusing TREM2 ECD with human DAP12 (aa28-113). The mutation D50A was introduced into DAP12 to eliminate the association with other receptors, allowing the constant cell surface expression of TREM2-DAP12 [25, 45]. oAβ significantly activated human TREM2 as indicated by the strong GFP signal (Supplementary Fig. 2f). In addition, both PS and PC showed strong activation of TREM2 NFAT reporter cells which confirm that PS and PC are ligands for TREM2 (Supplementary Fig. 2g). Notably, we found that only the oligomer Aβ activates LILRB2 and TREM2 reporter cells (Supplementary Fig. 2h). This observation is consistent with previous reports that LILRB2 and TREM2 only bind to oAβ but not the monomer Aβ [16, 20, 24].

Since TREM2 and LILRB2 are both expressed in microglia, and oAβ and PS have shared ligands that bind to both LILRB2 and TREM2, we next asked whether LILRB2, a receptor with ITIM motifs, may inhibit TREM2 signaling when the two receptors co-ligated as mediated by the shared ligands. An NFAT-GFP reporter system that simultaneously expresses both LILRB2 and TREM2 was constructed. A parental NFAT-GFP reporter cell expressing TREM2 was first generated. Then, two different constructs were introduced to engineer LILRB2-expressing reporter cells. In the reporter cell line named LILRB2-TREM2, the WT LILRB2 was introduced with fully functional ITIM motifs, which can mediate inhibitory signaling (Fig. 2e). A reporter cell line named LILRB2_muITIM-TREM2 was generated by introducing the full-length LILRB2 gene bearing three mutations (Y533F, Y562F, and Y592F) to abolish inhibitory functions of the ITIM motifs (Supplementary Fig. 2i). Both oAβ and PS induced strong inhibition of TREM2 signals in reporter cells co-expressing LILRB2 and TREM2 (Fig. 2f-g). Notably, with mutated ITIM (Y533F, Y562F, and Y592F), LILRB2 showed almost no inhibition of TREM2 signaling in comparison to TREM2-only reporter cells stimulated by ligands (Fig. 2f-g). Taken together, these results indicate that the shared ligands oAβ and PS induced inhibitory signals against TREM2 mediated by LILRB2, and the inhibition is dependent on the ITIM signaling of LILRB2.

We also validated the negative regulation of phagocytosis by LILRB2 using mouse microglia cell line BV2 overexpressing LILRB2. BV2 is widely used as a model microglia cell line to study the functions of receptor-of-interest in oAβ phagocytosis [24, 46, 47]. Upon introduction of LILRB2 into BV2 via lentivirus, we confirmed the expression of LILRB2 on the cell surface by flow cytometry (Supplementary Fig. 2j-k). In addition, BV2 showed spontaneous expression of mouse TREM2 by flow cytometry (Supplementary Fig. 2j-k). Introducing the WT LILRB2 into BV2 reduced oAβ-lipoprotein complex phagocytosis by 33.58% in comparison to BV2 expressing LILRB2 with dysfunctional ITIM (mutations Y533F, Y562F, and Y592F abolishing the tyrosine-mediated phosphatase recruitment of LILRB2) (Fig. 2h). The effects are consistent with the observation that LILRB2-mediated inhibition of TREM2 signaling is dependent on the ITIM motifs. We observed that the expression levels of TREM2 were similar between LILRB2 and LILRB2_muITIM, which rules out the possibility that the differences observed in phagocytosis are caused by differences in receptor expression levels (Supplementary Fig. 2k). To validate the increased phagocytosis signal was a result of oAβ phagocytosis, the above-mentioned treatment groups were repeated side by side by co-incubation with CytoD, a reagent selectively inhibiting phagocytosis by blocking actin-cofilin interaction [48]. As shown in Fig. 2h, the inclusion of CytoD in the treatment abolished all the intergroup differences in oAβ-lipoprotein complex phagocytosis observed when CytoD was absent. With CytoD treatment, the phagocytosis levels dropped to about 15% of the level in the Ctrl-BV2 cells when CytoD was not present.

### LILRB2 antibodies block LILRB2 signaling triggered by oAβ and PS

As demonstrated in the results shown in Fig. 2, ligands shared by LILRB2 and TREM2, such as oAβ and PS, can inhibit TREM2 signaling by inducing the co-ligation between LILRB2 (an inhibitory receptor with ITIM motifs) and TREM2 (an activating receptor delivers signals through ITAM motifs of DAP12). To rescue the TREM2 signaling blockade caused by co-ligation with LILRB2, we next sought to develop antibodies to block oAβ and PS from binding to LILRB2. Since the D1 and D2 domains of LILRB2 are crucial to the ligand binding, we panned the phage-displayed scFv antibody libraries against the D1 and D2 domains of LILRB2 for mAbs that block ligand-receptor interaction. Among the D1-D2 domain-specific scFv clones showing the highest binding in phage ELISA against LILRB2-Fc, we selected 21 unique mAb sequences and converted them into human IgG1 for further functional screening of antagonistic antibodies that block oAβ and PS-induced LILRB2 signaling.

**Fig. 3:**
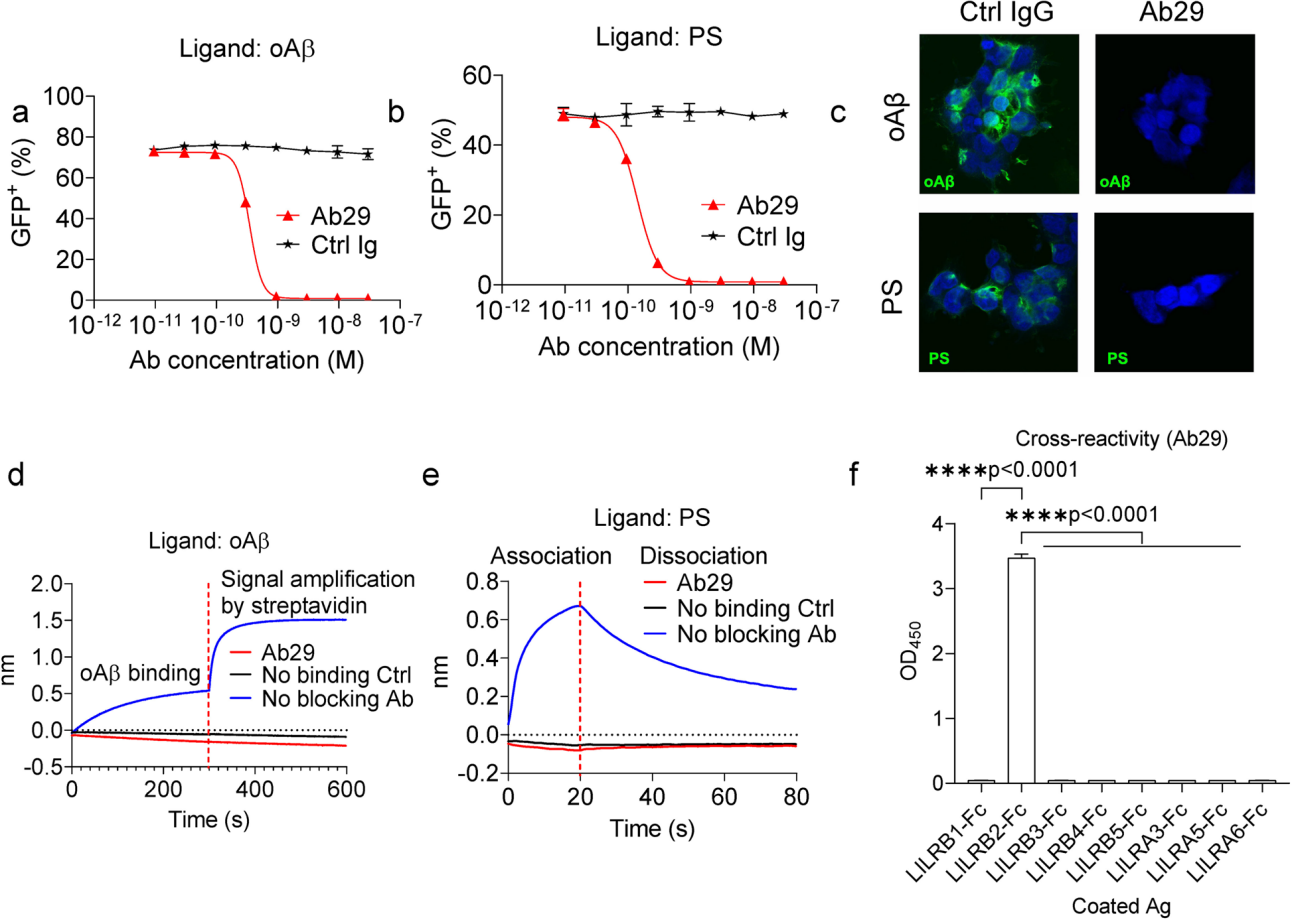
LILRB2 targeting antibody Ab29 showed potent blocking activity, high affinity, and specificity. **a**-**b**. Titration of blocking the activity of Ab29 against oAβ or PS-LILRB2 interactions. Plate-coated oAβ (**a**) or PS (**b**) was incubated with LILRB2-chimeric reporter cells under the presence of increasing concentrations of Ab29 or control IgG. The activation of LILRB2-chimeric reporter cells was observed as a percentage of GFP^+^ cells. Data are presented as mean ± SD (*n* = 3 independent experiments). **c**. Representative immunostaining images showing Ab29 blocks oAβ and PS from binding to LILRB2 expressed on the cell surface. Scale bar represents 5 μm. **d**. Ab29 blocks LILRB2 binding with oAβ as measured by BLI. LILRB2 was loaded onto protein A sensors via binding with sensor-captured Ab29. The LILRB2-loaded sensors were then incubated with biotinylated oAβ (1 μM) and the binding signals were amplified with streptavidin. The amount of oAβ bound onto the sensors was presented as wavelength shift in nanometers (nm). The red dotted vertical line marks the transit from incubation with biotinylated oAβ to signal amplification by streptavidin. **e**. Ab29 blocks LILRB2 binding with PS liposomes as measured by BLI. LILRB2 was loaded onto protein A sensors via binding with sensor-captured Ab29. The LILRB2-loaded sensors were incubated with PS liposomes (1 mM) (association stage) and then the sensors were dipped into kinetics buffer without PS allowing the bound PS to freely dissociate (dissociation stage). The amount of PS bound onto the sensors was presented as wavelength shift in nanometers (nm). The red dotted vertical line marks the transit from association stage to dissociation stage. **f**. Cross-reactivity of Ab29 against other LILRB and LILRA family receptors as measured by ELISA. Plate-coated LILRB and LILRA family receptors (shown in the x-axis) were incubated with Ab29 (4 nM). The amount of bound antibody was detected by anti-human F(ab)2 HRP, and the values are shown as OD_450_. LILRB2-Fc was included as the positive control. Data are presented as mean ± SD (*n* = 4 independent experiments)

For reporter assay using oAβ as the ligand, blocking activities were observed for all 21 antibodies as indicated by the decreased GFP^+^ percentage over the control (Supplementary Fig. 3a). Similarly, for reporter assay using PS as the ligand, blocking effects were observed for 19 of the 21 antibodies (all antibodies except Ab8 and Ab128) as indicated by the decreased GFP^+^ percentage (Supplementary Fig. 3b). Within the 21 antibodies, 11 antibodies showed complete blocking of both oAβ and PS-induced LILRB2 signaling (Supplementary Fig. 3a-b). The 11 antibodies were then purified by protein A chromatography and further titrated using the chimeric LILRB2 NFAT-GFP reporter assay. As shown in Supplementary Fig. 3c-d and Supplementary Tables 1 and 2, 6 out of 11 and 11 out of 11 antibodies have IC_50_ less than 10 nM in assays using oAβ or PS as the ligand, respectively. Within all the antibody candidates, Ab29 showed the strongest blocking of LILRB2 signaling induced by both ligands (Fig. 3a-b). Immunocytochemistry also confirmed that Ab29 blocks both oAβ and PS from binding to LILRB2 expressed on the cell surface of HEK293Tcells (Fig. 3c).

We further studied Ab29 on its ligand blocking, kinetics profile, cell surface LILRB2 binding, and cross-reactivity. As shown in Fig. 3d, Ab29-captured LILRB2 showed no binding to oAβ as indicated by the similar binding signals to the no-binding control (with no LILRB2-loaded onto the sensors). The absence of signals indicates competition of LILRB2 binding by Ab29 and ligand oAβ. In contrast, control Ab showed no blocking of oAβ binding with LILRB2 (Fig. 3d and Supplementary Fig. 3e). A similar BLI assay was designed for Ab29 blocking of PS binding with LILRB2. As shown in Fig. 3e, Ab29-captured LILRB2 showed no binding to PS. In comparison, the control Ab did not inhibit PS-LILRB2 interactions (BLI signals 0.67 vs. baseline-level signals from control IgG, Supplementary Fig. 3f). In addition to Ab29, we studied all other purified antibody candidates for blocking the interactions between LILRB2 and oAβ or PS using BLI. As expected, all antibodies except Ab37 showed complete blocking of interactions between LILRB2 to oAβ and PS (Supplementary Fig. 3g-h). Taking together, the BLI results confirmed that LILRB2 blocking antibodies function by directly blocking the interactions between LILRB2 and its ligand oAβ or PS.

We next characterized the biophysical properties of Ab29. Ab29 demonstrated a fast association to LILRB2 (kon = 1.68 × 10^5^ 1/Ms) and slow dissociation from LILRB2 (kdis = 1.51 × 10^− 3^ 1/s), with an affinity constant K_D_ = 9 nM (Supplementary Fig. 3i). Ab29 and Ab37 exhibited the highest binding affinities to LILRB2 among the 11 antibodies, but Ab29 possessed the slowest dissociation rate (Supplementary Table 3). The high-affinity binding and, more importantly, the slow antigen dissociation rate explain that Ab29 is the most potent antibody in blocking ligand-activated LILRB2 signaling. ELISA titration against plate-coated LILRB2 antigen showed strong Ab29 binding to LILRB2 with EC_50_ = 0.14 nM (95%CI 0.13-0.15 nM) (Supplementary Fig. 3j). Ab29 and Ab36 were the two highest binders among the 11 antibodies tested (Supplementary Fig. 3j and Supplementary Table 4). In addition to ELISA titration and BLI assays, Ab29 also demonstrated specific binding to LILRB2-expressing HEK293T (Supplementary Fig. 3k). Except for Ab37, the other nine antibodies were also found to bind specifically to LILRB2-expressing HEK293T instead of the parent HEK293T cells. Ab37 showed non-specific binding to HEK293T cells (Supplementary Fig. 3k) and thus is not an ideal candidate even though it has shown strong blocking effects (Supplementary Fig. 3c-d). Taken together, multiple assays were employed to select Ab29 as the top antibody candidate with desired biophysical and functional properties.

Due to the high similarity between LILRB2 and other receptors in the LILRB and LILRA families, we also tested the cross-reactivity of LILRB2 antibodies against 10 receptors in the LILR family using both BLI and ELISA assays. In the ELISA assay, all 11 LILRB2 antibodies including Ab29 showed no cross-reactivity to the rest of the LILR family of receptors (Fig. 3f and Supplementary Fig. 3l). In the BLI assay, Ab29 showed high specificity against LILRB2 as it exhibited no binding to the rest of LILRB receptors and LILRA receptors (Supplementary Fig. 3m, binding signals 1.44 for LILRB2 vs. baseline-level signals as shown in Supplementary Fig. 3n).

### Ab29 potently inhibits LILRB2-TREM2 co-ligation

**Fig. 4:**
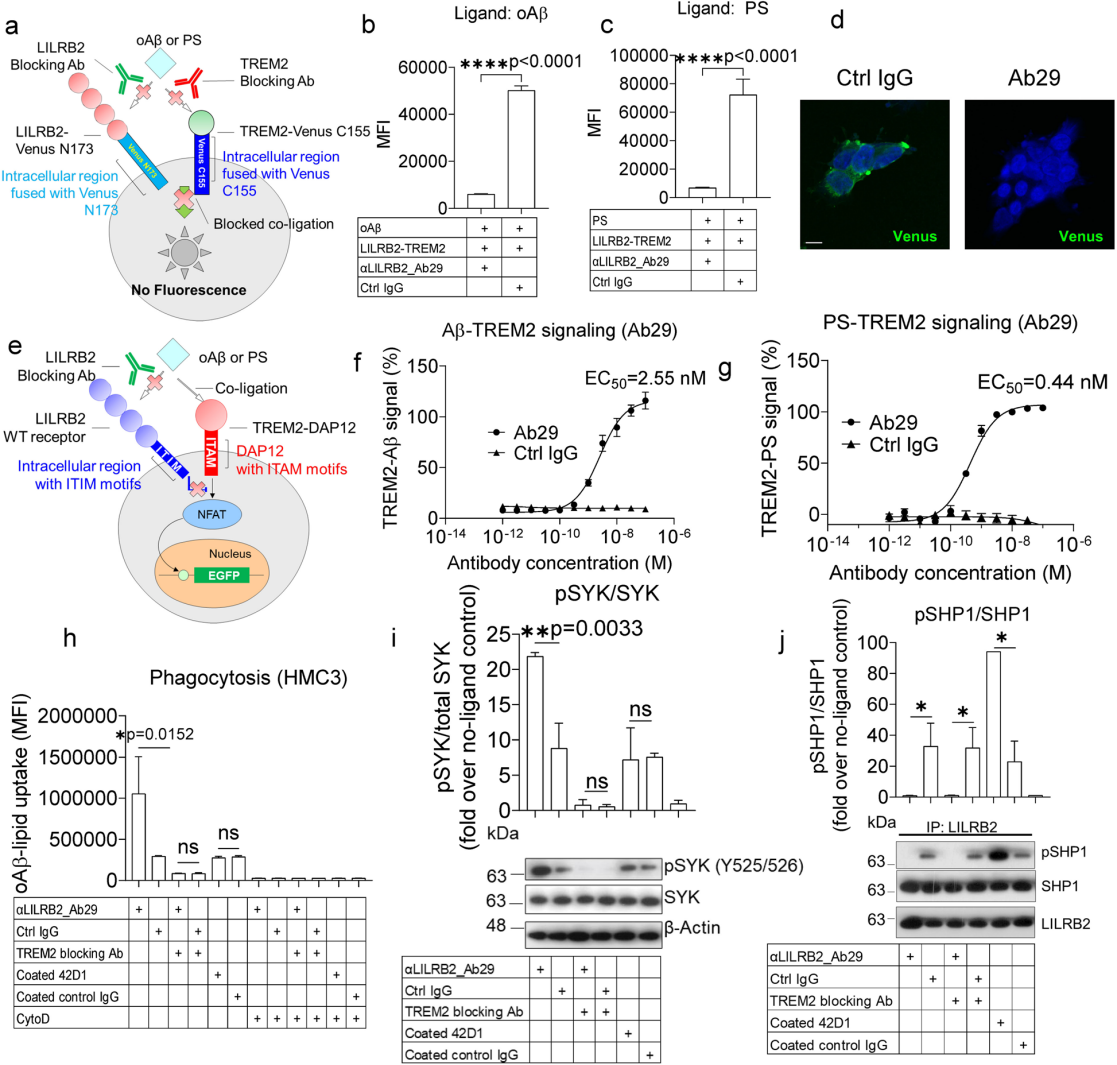
*LILRB2* blocking antibody Ab29 completely rescued LILRB2-mediated TREM2 signaling inhibition and promoted microglial phagocytosis by blocking LILRB2/TREM2 co-ligation. **a**. Schematic diagram showing the design of a BiFc assay studying the effect of LILRB2 and TREM2 blocking antibodies in preventing the ligand-mediated receptor co-ligation. **b** and **c**. LILRB2 blocking antibodies abolish oAβ or PS-induced co-ligation of LILRB2 and TREM2. Antibodies at 10 μg/mL were incubated with HEK293T cells co-expressing LILRB2-N173 Venus and TREM2-C155 Venus plus plate-coated oAβ (**b**) or PS (**c**). The MFI signals from complemented Venus are shown. Data are presented as mean ± SD (*n* = 3 independent experiments). **d**. Representative immunofluorescence images showing Ab29 blocks oAβ-lipid from inducing LILRB2-TREM2 co-ligation. Scale bar represents 5 μm. **e**. schematic diagram showing LILRB2 blocking antibody rescues TREM2 signaling inhibition by blocking LILRB2-ligand interactions. The assay is depicted as an NFAT-GFP reporter assay. The ITIM motifs of LILRB2 block signaling generated by ITAM motifs of TREM2 upon cross-linking of the two receptors by shared ligands oAβ or PS. **f**-**g**. Titration of Ab29 in rescuing of oAβ or PS-LILRB2-induced inhibition of TREM2 signaling. Plate-coated oAβ (**f**) or PS (**g**) was incubated with LILRB2/TREM2 reporter cells under the presence of increasing concentrations of Ab29 or control IgG. The activation of LILRB2/TREM2 reporter cells was observed as percentages of GFP^+^ cells. TREM2 signaling of treatment groups was normalized based on the percentage of GFP^+^ reporter cells expressing only TREM2 (set to 100%). Data are presented as mean ± SD (*n* = 4 independent experiments). **h**. Ab29 promotes oAβ-lipoprotein complex phagocytosis by HMC3 human microglial cell line. HMC3 cells were incubated with FAM-oAβ-lipoprotein complex and indicated antibodies (treatment table). Phagocytosis was quantified by flow cytometry, with the MFI shown. Negative phagocytosis control included 10 μM CytoD together with indicated treatments. Data are presented as mean ± SD (*n* = 4 independent experiments). **i**. Ab29 improves TREM2 pathway signaling as indicated by the increased pSYK level. HMC3 cells were incubated with oAβ-lipoprotein complex with indicated treatments for 1 hour. Immunoblot of phosphorylated SYK (pSYK), total SYK of HMC3 upon treatment, with β-actin as the loading control. Data are presented as mean ± SD (*n* = 3 independent experiments). **j**. Ab29 blocks LILRB2 pathway signaling as indicated by the decreased pSHP1 level. Immunoblot of phosphorylated SHP1 (pSHP1), SHP1 of HMC3 upon incubation with oAβ-lipoprotein complex with indicated treatments for 1 hour. Results are from anti-LILRB2 immunoprecipitation with LILRB2 as the loading control. Data are presented as mean ± SD (*n* = 3 independent experiments)

After establishing that Ab29 can block LILRB2-ligand interactions, we next asked whether Ab29 can inhibit the co-ligation of LILRB2 and TREM2, thus preventing the LILRB2-mediated TREM2 signaling inhibition. We first studied whether Ab29 blocks LILRB2 and TREM2 co-ligation using the BiFc assay as shown in Fig. 4a. The Ab29 showed complete blocking of oAβ- or PS-mediated LILRB2-TREM2 co-ligation (Fig. 4b-d).

Using the NFAT-GFP reporter assay shown in Fig. 4e, antibodies were tested for their ability to restore the LILRB2-mediated TREM2 signaling inhibition. Using oAβ as the ligand, reporter cells co-expressing WT LILRB2 and TREM2 (LILRB2-TREM2) were treated with the antibodies at a fixed concentration of 10 μg/mL. When compared to the antibody-treated reporter cells expressing only TREM2, the 11 tested antibodies showed a varying degree of rescuing of TREM2 signaling, and Ab29 and Ab37 showed complete rescue of TREM2 signaling (Supplementary Fig. 4a). Using PS as the ligand at the fixed concentration of 10 μg/mL, 5 antibodies (Ab29, Ab37, Ab55, Ab60, and Ab93) showed complete rescue of TREM2 signaling (Supplementary Fig. 4b). Since Ab29 was the most potent antibody, it was further titrated for blocking oAβ- and PS-induced and LILRB2-mediated TREM2 signaling inhibition with EC_50_ = 2.55 nM (Fig. 4f) and 0.44 nM (Fig. 4g), respectively.

TREM2 deficiency has shown a direct impact on Aβ clearance by microglia [42]. It was reported previously that TREM2 binds to lipoproteins including low-density lipoprotein (LDL); clusterin (CLU), also known as apolipoprotein J (APOJ); and apolipoprotein E (APOE), and promotes Aβ-complexed lipoproteins uptake by microglia [42]. Macrophages with TREM2 loss-of-function variant R47H show reduced ligand binding and phagocytosis of the Aβ-lipoprotein complex [42]. We assessed whether LILRB2-mediated TREM2 signaling inhibition affects the microglia phagocytosis of the oAβ-lipid complex and whether LILRB2-blocking antibodies can reverse the effects. Using an immortalized human microglial cell line HMC3, we studied the phagocytosis of the oAβ-lipid complex upon LILRB2 blocking antibody treatment.

We first confirmed the spontaneous expression of LILRB2 and TREM2 on the HMC3 cell surface (Supplementary Fig. 4c-d). Ab29 treatment significantly increased phagocytosis in comparison to control IgG (Fig. 4h). We next tested the effect of Ab29 on SYK phosphorylation, which is the key component of the TREM2-DAP12 signaling pathway that regulates microglia-mediated oAβ phagocytosis. Ab29-treated HMC3 cells showed a significant increase in pSYK/SYK ratios over Ctrl IgG-treated cells (Fig. 4i). Moreover, we studied whether Ab29-treatment blocks LILRB2 signaling. SHP1 phosphorylation associated with LILRB2 is the key component of the LILRB2 signaling pathway [15]. Ab29-treated HMC3 cells showed a significant decrease in LILRB2-associated pSHP1/SHP1 ratio over Ctrl IgG-treated cells (Fig. 4j). In contrast, the Ab29 treatment showed no significant changes in the overall pSHP1/SHP1 ratios over Ctrl IgG-treated cells (Supplementary Fig. 4e), indicating the specific effects on LILRB2 by Ab29.

We then studied whether the effects of LILRB2 Ab29 are dependent on TREM2 signaling using a commercially available TREM2 antagonist antibody. The TREM2 antagonist antibody blocks the TREM2 signaling induced by both oAβ and PS in both mouse and human TREM2 reporter cells (Supplementary Fig. 4f-i). Co-incubation of LILRB2 Ab29 with TREM2 antagonist antibody abolished the significant differences in oAβ phagocytosis between Ab29-treated and Ctrl IgG-treated HMC3 cells (Fig. 4h). In addition, TREM2 antagonist treatment abolishes the differences in SYK phosphorylation induced by the Ab29 treatment (Fig. 4i). The observation in SYK phosphorylation again indicates that Ab29-mediated effects are dependent on the TREM2 signaling pathway.

We next tested whether activating LILRB2 alone would inhibit microglia phagocytosis by using a plate-bound non-blocking anti-LILRB2 antibody clone 42D1 to crosslink and activate LILRB2 [49]. Antibody clone 42D1 was validated to show no antagonistic effects in LILRB2 signaling induced by either oAβ or PS using the LILRB2 reporter cells (Supplementary Fig. 4j). The plate-bound 42D1 was also verified to induce LILRB2 signal activation in LILRB2 reporter cells (Supplementary Fig. 4k). We observed increased SHP1 phosphorylation induced by plate-bound anti-LILRB2 clone 42D1 (Fig. 4j). However, neither the pSYK/SYK ratios nor the oAβ phagocytosis showed significant differences by plate-bound anti-LILRB2 clone 42D1 (Fig. 4h-i). Anti-LILRB2 clone 42D1 treatment did not stimulate oAβ phagocytosis when compared to the coated control IgG (Fig. 4h), which indicates that activation of LILRB2 alone without crosslinking with TREM2 did not affect phagocytosis in microglia cells. In addition, anti-LILRB2 clone 42D1 treatment did not alter the pSYK/SYK ratio when compared to the coated control IgG (Fig. 4i), which indicates that activation of LILRB2 alone without crosslinking with TREM2 did not affect pSYK/SYK ratio in microglia cells. Since we correlate the pSYK/SYK ratio with TREM2 activation, these data indicate that the activation of LILRB2 alone without crosslinking with TREM2 did not induce inhibition in TREM2 signaling in microglia. The commercial anti-LILRB2 42D1 was additionally tested and found not to rescue LILRB2-mediated TREM2 inhibition as shown in Supplementary Fig. 4l. This is consistent with the observation described above that 42D1 does not block LILRB2 interactions with ligands as shown in Supplementary Fig. 4j.

### Ab29 potently enhance microglia functions in phagocytosis, migration, and cytokine responses

**Fig. 5:**
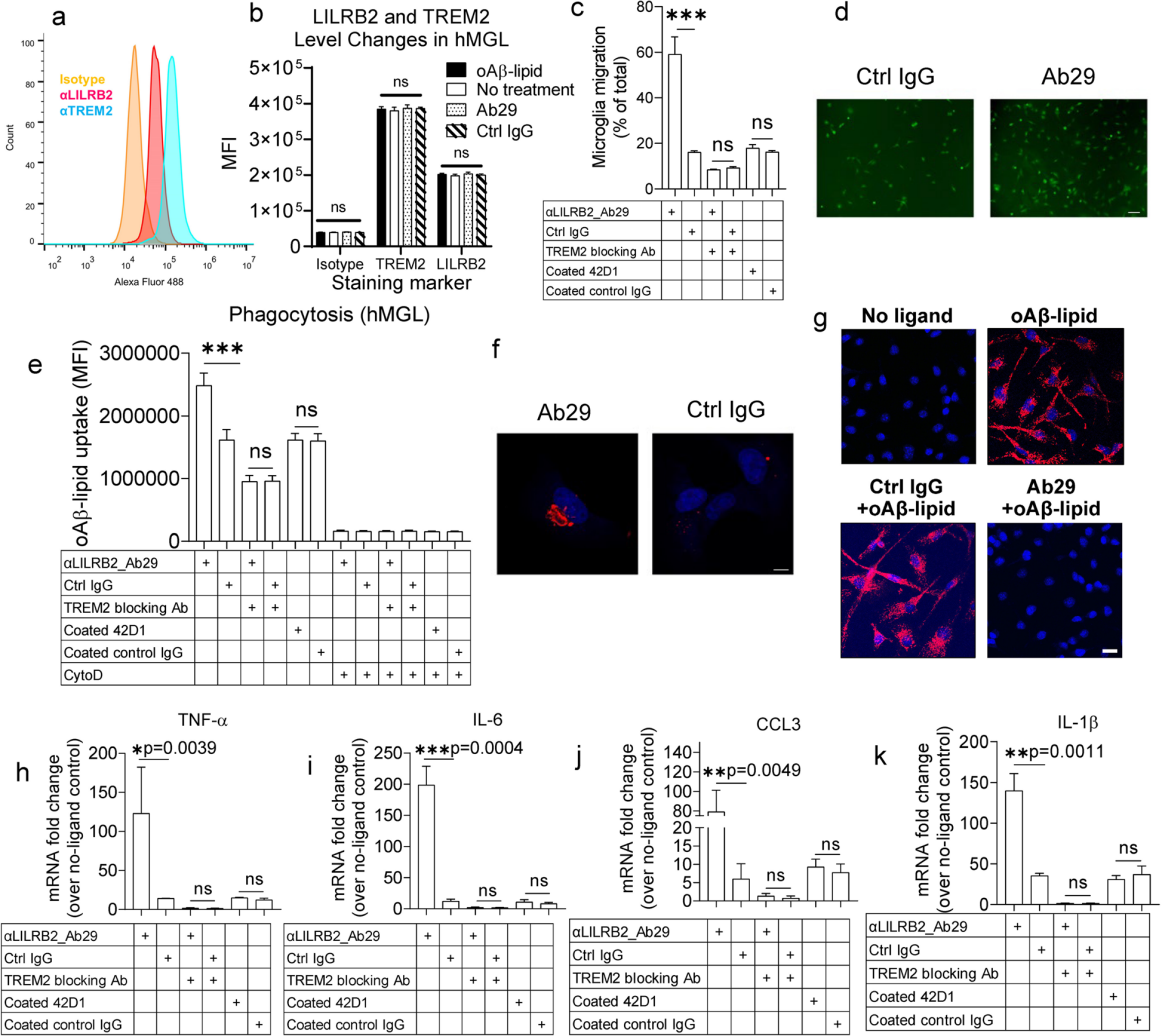
LILRB2 blocking antibody Ab29 promoted hMGL migration, phagocytosis, and cytokine responses. **a**. Surface expression of TREM2 and LILRB2 on hMGL cells. The fluorescent signals (x-axis) were plotted as a histogram with normalized cell percentage in the y-axis. **b**. MFI of hMGL cells labeled by indicated antibodies (x-axis) as shown in a. Data are presented as mean ± SD (*n* = 3 independent experiments). **c**. Ab29 promotes hMGL cell migration. hMGL cells were plated onto transwell chamber inserts, and hMGL cell migration toward the oAβ-lipoprotein complex in the receiving chamber was measured with treatments shown. For quantification, crystal violet-stained hMGL cells were quantified by eluting crystal violet in 33% acetic acid and measured the absorbance at 590 nm using a plate reader. Percentages of migrated cells over total are plotted. Data are presented as mean ± SD (*N* = 3 independent experiments). **d**. Representative immunostaining images showing Ab29 promotes hMGL migration induced by oAβ-lipid. The hMGLs were pre-labeled with CFSE to visualize the cells under the fluorescence microscope. Scale bar = 100 μm. **e**. Ab29 promotes oAβ-lipoprotein complex phagocytosis by hMGLs. hMGL cells were incubated with FAM-oAβ-lipoprotein complex and indicated antibodies. Phagocytosis was quantified by flow cytometry, with the MFI shown. Negative phagocytosis control included 10 μM CytoD together with indicated treatments. Data are presented as mean ± SD (*n* = 3 independent experiments). **f**. Representative immunostaining images showing Ab29 promotes hMGL phagocytosis of oAβ-lipid. Scale bar represents 5 μm. **g**. PLA assay indicates that oAβ-lipid induces clustering of LILRB2 and TREM2, while Ab29 blocks the clustering. hMGLs were incubated with oAβ-lipid (100 nM) and antibodies (Ctrl IgG or Ab29, 10 μg/mL), and the LILRB2 and TREM2 clustering were detected by PLA assay. The red color indicates the PLA signals detected, and the blue color represents the cell nucleus. Scale bar = 20 μm. **h**-**k**. Ab29 promotes oAβ-lipoprotein complex-induced inflammatory cytokine responses in hMGLs. hMGL cells were incubated with oAβ-lipoprotein complex with indicated treatments. The relative mRNA levels of TNF-α (**g**), IL-6 (**h**), CCL3 (**i**), and IL-1β (**j**) were determined using qRT-PCR with GAPDH as the internal control. Data are presented as mean ± SD (*n* = 3 independent experiments)

TREM2 has been shown to regulate various key functions of microglia, such as cytokine levels, migration toward β-amyloid plaques, and phagocytosis of β-amyloid [24]. Microglia migration is crucial in clearing β-amyloid plaques by clustering of microglia around β-amyloid plaque [24]. Pro-inflammatory cytokines from microglia are crucial in regulating brain inflammatory responses against β-amyloid pathology [24]. Due to the lack of mouse LILRB2 homolog with high sequence similarity, we next studied whether Ab29 can enhance the microglia functions including migration, phagocytosis, and cytokine response upon oAβ-lipid stimulation in human induced pluripotent stem cell (iPSC)–derived microglia (hMGLs). hMGLs are widely used to model human microglia due to the high similarities to in vivo microglia in cytokine, migration, and phagocytosis response to oAβ [24, 29–31]. We first validated the spontaneous expression of LILRB2 and TREM2 on the hMGL cell surface (Fig. 5a-b). Also, as shown in Fig. 5b, oAβ-lipid incubation with hMGLs or treatments with LILRB2 Ab29 did not change the cell surface levels of LILRB2 and TREM2 as measured by flow cytometry.

Ab29 treatment significantly increased hMGL migration toward oAβ-lipid complexes in comparison to control IgG (Fig. 5c-d). Ab29 treatment significantly increased phagocytosis in comparison to control IgG (Fig. 5e-f). CytoD treatment was validated as an appropriate negative control for phagocytosis by comparing it to the phagocytosis of hMGLs incubated at 4 °C. Notably, as shown in Supplementary Fig. 4m, CytoD treatment reduced the observed fluorescence signals to a similar level to 4 °C incubations, indicating the differences observed between treatments with and without CytoD were truly phagocytosis. Additionally, in our experiment, the cell surface-bound oAβ-FAM signals were quenched with trypan blue, which is also confirmed (Supplementary Fig. 4m). The combination of CytoD with trypan blue reduced the fluorescence signals to a level similar to unstained cells, fully validating the procedures.

We next validated the clustering of LILRB2 and TREM2 in hMGLs using PLA assay. PLA assay has been widely used as a trusted approach to visualize the proximity clustering of proteins [50, 51]. As shown in Fig. 5g, when hMGLs were incubated with oAβ-lipid, proximity ligation between LILRB2 and TREM2 was observed clearly in comparison to the control group without the ligand. More importantly, when we co-incubated oAβ-lipid with Ab29, the proximity ligation between LILRB2 and TREM2 was abolished, while Ctrl IgG did not show such effects. Overall, we conclude that oAβ-lipid induces the proximity ligation between LILRB2 and TREM2, which is effectively blocked by Ab29.

Next, we studied the effect of Ab29 treatment on the expression of pro-inflammatory cytokines TNF-α, IL-6, CCL3, and IL-1β. Pro-inflammatory cytokines from microglia are crucial in regulating brain inflammatory responses against β-amyloid pathology [24]. oAβ has been shown to upregulate pro-inflammatory cytokines in microglia [24]. Ab29 treatment significantly increased the mRNA levels of proinflammatory cytokines in comparison to control IgG (Fig. 5h-k). TREM2 antagonist abolished the Ab29-induced increase of hMGL migration (Fig. 5c). Also, blocking TREM2 abolished the enhanced oAβ phagocytosis in hMGL induced by the Ab29 treatment (Fig. 5e). In addition, TREM2 blockade abolished the increase of pro-inflammatory cytokine expression induced by Ab29 (Fig. 5h-k). The observations that TREM2 blockade abolishes the improvement by LILRB2 antagonism indicate that LILRB2 is upstream of TREM2 in regulating hMGL biological functions.

Plate-bound anti-LILRB2 clone 42D1 showed no significant differences in hMGL migration in comparison to Ctrl IgG, which indicates activation of LILRB2 alone without crosslinking with TREM2 does not inhibit hMGL migration (Fig. 5c). LILRB2 activation alone by plate-bound anti-LILRB2 clone 42D1 showed no significant differences in oAβ phagocytosis in hMGL (Fig. 5e). In addition, LILRB2 activation alone did not increase pro-inflammatory cytokine expression (Fig. 5h-k). Plate-bound anti-LILRB2 clone 42D1 showed no significant differences in hMGL migration, phagocytosis, and cytokine response in comparison to Ctrl IgG, which indicates the effects of LILRB2 on hMGL require co-ligation with TREM2.

### Blocking LILRB2 promotes microglial phagocytosis of amyloid plaques in 5XFAD mice

**Fig. 6:**
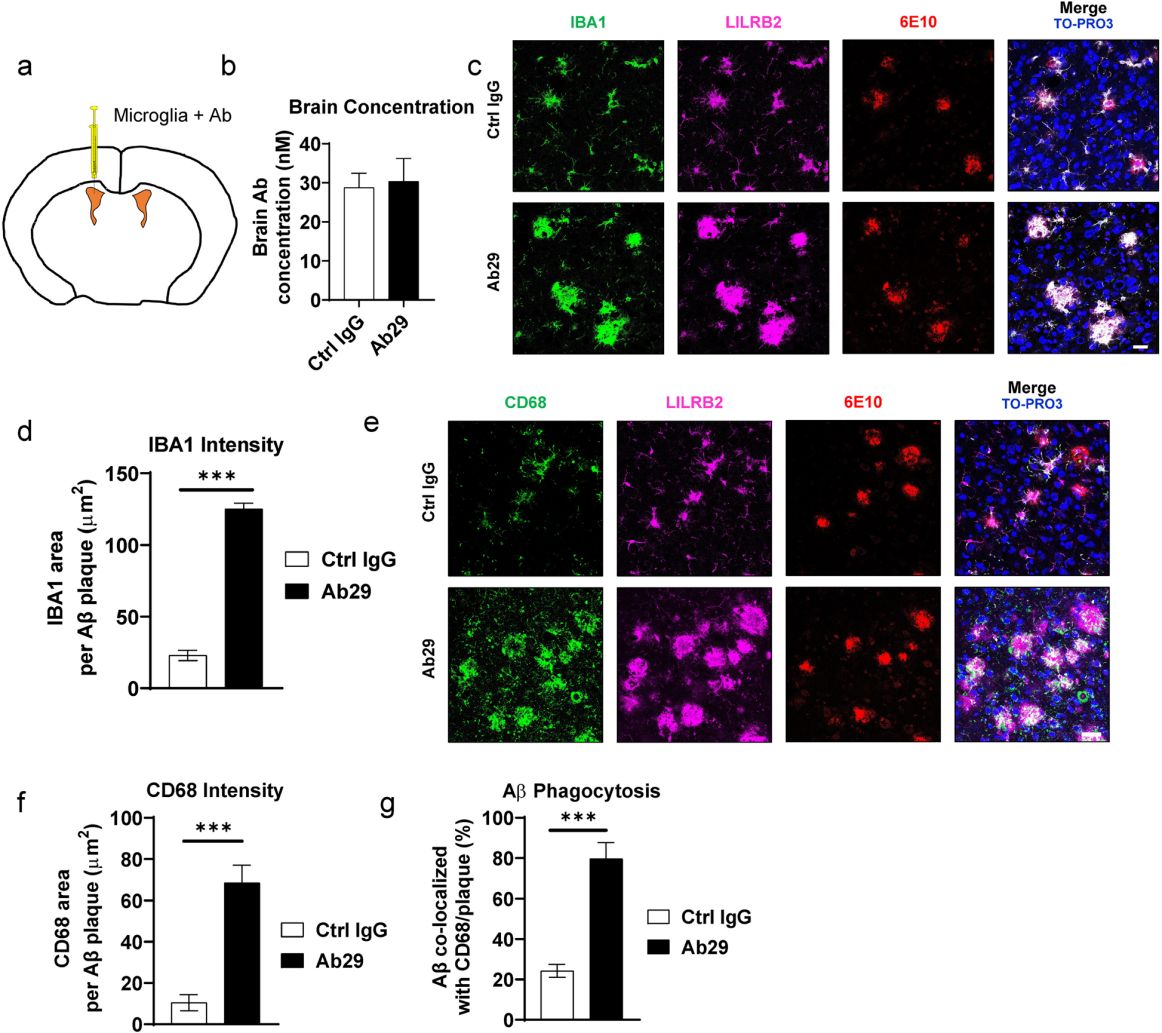
Ab29 increases microglial responses to amyloid plaques in vivo. **a**. schematic diagram showing the stereotaxic injection of hMGLs and Ab29 in 5XFAD mice. The hMGLs were injected into the lateral ventricles through stereotaxic injections together with antibodies. The 5XFAD mice were sacrificed 96 hours after the injection and brains were harvested for analysis. **b**. Antibody concentrations in perfused 5XFAD at the experiment endpoint as described in a. *n* = 5 independent mice. **c**. Representative amyloid plaque-microglia co-localization immunofluorescence staining of 5-month-old 5XFAD mice cortex as treated in a. Scale bar = 20 μm. IBA1, microglia marker; 6E10, amyloid plaque marker; the nucleus is labeled by TO-PRO-3. **d**. Quantification of IBA1 area within 30 μm of amyloid plaques in the cortex of mice treated as described in a. *n* = 5 independent mice. **e**. Representative amyloid plaque-CD68 co-localization immunofluorescence staining of the cortex of 5XFAD mice treated as described in a. CD68, microglia phagocytic marker; the nucleus is labeled by TO-PRO-3. Scale bar = 20 μm. **f**. Quantification of CD68 area within 30 μm of amyloid plaques in the cortex of mice treated as described in a. *n* = 5 independent mice. **g**. Quantification of Aβ co-localized with CD68 per plaque in the cortex of mice treated as described in a. *n* = 5 independent mice. For all the data presented, bar graphs with error bars represent mean ± SD. For the statistical analysis, *** *P* < 0.001, two-tailed Student t-test

As demonstrated above, Ab29 blocks LILRB2 and promotes oAβ-lipid phagocytosis by microglia, and improves microglia migration toward oAβ-lipid. We next validated the effect of Ab29 on microglial response to amyloid plaques in 5XFAD mice, which is an established AD mouse model that develops spontaneous amyloid plaques. Because mice do not have LILRB2, we introduced hMGLs through implantation. Implantation of hMGLs has been reported before and the in vivo behavior of implanted hMGLs is proven to be similar to native microglia [30]. We used 5-month-old 5XFAD mice, which have already accumulated sufficient amyloid plaques, for the experiment [35, 52]. As shown in Fig. 6a, the hMGLs were implanted into bilateral ventricles, which allow the cells to migrate to other brain regions [33]. The 4d short-term treatment is designed to avoid complications of long-term grafting rejection of human cells by the mice. Although short, the 4d time period is sufficient for microglial migration to the cortex through the lateral ventricles [53]. We administered the antibodies only once together with the implanted hMGLs. A single intracerebroventricular (ICV) injection maintained an effective concentration of antibodies of above 30 nM inside the brain at the end of experiment (Fig. 6b), which is 10-fold more than the EC_50_ value of 2.55 nM shown in Fig. 4f.

To study the co-localization of microglia with Aβ plaques, we collected the brains after perfusion and floating slices were stained by 6E10 to label Aβ plaques. For microglia, we used human IBA1-specific, human LILRB2-specific, and human nuclei-specific antibodies to label human microglia. IBA1 signals within 30 μm of plaques showed a significant increase of 5-fold in the Ab29 treated mice over the Ctrl IgG group (Fig. 6c and d, Supplementary Fig. 5a and b), suggesting that Ab29 significantly increased microglia clustering around plaques. This observation is consistent with our studies in vitro showing Ab29-mediated LILRB2 blocking promotes microglia migration toward the oAβ-lipid complex. Immunostaining also confirmed the co-localization of LILRB2 with IBA1, which is consistent with the fact that hMGLs express LILRB2.

In addition to the clustering of microglia around plaques, the phagocytosis of microglia is also crucial in the removal of amyloid. CD68, the phagocytic marker of microglia, is frequently used to study the phagocytic status of plaque proximal microglia [35, 54, 55]. We co-stained human CD68, human LILRB2, human nuclei, and plaques (6E10) and observed a significant increase of CD68 intensity around plaques in the Ab29-treated mice over the Ctrl IgG group (Fig. 6e and f, Supplementary Fig. 5c and d). In addition, we observed a significant (4.6-fold) increase of the co-localization between Aβ and CD68 (Fig. 6g, Supplementary Fig. 5e). This observation is consistent with our in vitro study showing Ab29-mediated LILRB2 blocking promotes microglia phagocytosis of the oAβ-lipid complex. We also observed co-localization of CD68 signals with LILRB2, which confim that both signals are from microglia. Taken together, the Ab29 treatment increased microglia clustering around amyloid plaques and the subsequent phagocytosis of plaques in vivo.

### Mapping key domains and residues of LILRB2 responsible for ligand binding

**Fig. 7:**
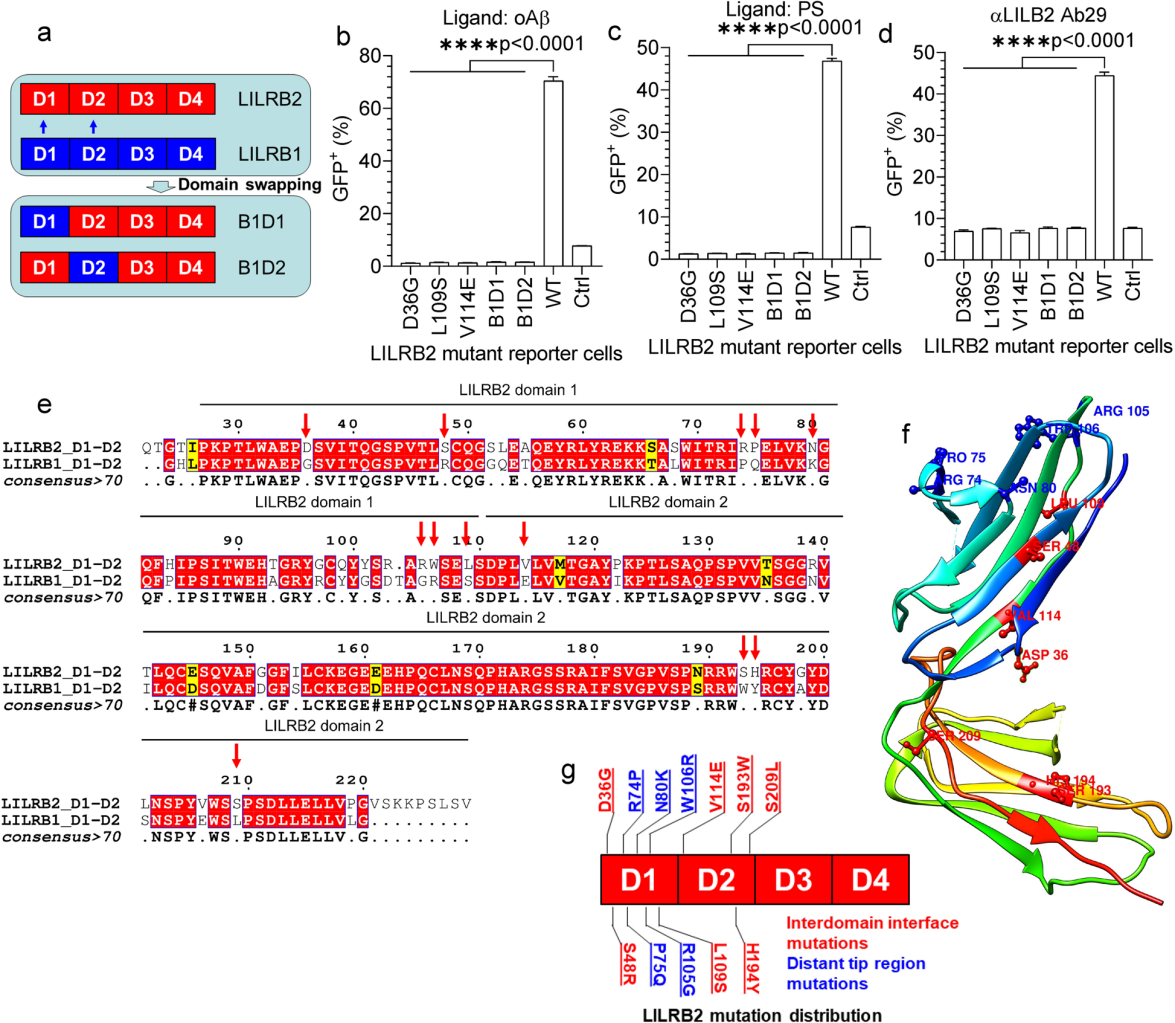
Key domains and amino acid residues of LILRB2 for ligands and antibody binding. **a**. Schematic diagram showing the design of domain-swapping mutants of LILRB2. B1D1 and B1D2 mutants were generated by replacing the D1 and D2 domains of LILRB2 with their corresponding domains from LILRB1, respectively. **b**-**d**. oAβ, PS, and Ab29 binding to LILRB2 mutants as measured on chimeric reporter cells. Chimeric NFAT-GFP reporter cells expressing individual mutants of LILRB2 (listed in x-axis) were incubated with oAβ (**b**) PS (**c**), and Ab29 (**d**). The activation of reporter cells is shown as the percentage of GFP^+^ cells. Data are presented as mean ± SD (*n* = 3 independent experiments). **e**. Alignment of D1 and D2 domains of LILRB2 and LILRB1. Amino acid residues are numbered according to Uniprot entry Q8N423. Identical residues are shown in white text with red background. Similar residues are shown in black text with a yellow background. Non-conserved residues are in white background. Individual domains of LILRB2 are also labeled. The red arrows indicate the single-amino acid mutations designed for testing. **f**. Crystal structure of LILRB2 (2gw5) visualized in UCSF Chimera showing the D1 and D2 domains as ribbons. Selected residues for mutation studies are shown in ball-and-stick with side chains displayed. Three-letter code names and residue numbers are also labeled following Uniprot entry Q8N423. **g**. Schematic diagram showing the distribution of single-amino acid mutations in LILRB2 D1 and D2 domains

Since D1 and D2 domains of LILRB2 are needed for ligand binding as shown in Fig. 1, we next studied the roles of individual domains (D1 and D2 domains) in ligand binding using a chimeric reporter cell assay similar to those demonstrated in Fig. 2. We first created two domain-swapping mutants of LILRB2: B1D1 was created by replacing the D1 domain of LILRB2 with the corresponding domain from LILRB1, and B1D2 was created by replacing the D2 domain of LILRB2 with the corresponding domain from LILRB1 (Fig. 7a). The expression level of both mutants was verified using anti-HA antibody 12CA5, showing a similar expression level to WT LILRB2 with HA tag (Supplementary Fig. 6a). Replacing either the D1 or D2 of LILRB2 with the corresponding domain of LILRB1 significantly reduced the binding between mutant LILRB2 and oAβ in comparison to WT LILRB2 (Fig. 7b). These results indicate that both the D1 and D2 domains contribute to the oAβ binding to LILRB2. The LILRB2 mutants with D1 or D2 replaced by the D1 or D2 of LILRB1 also showed significantly reduced binding signals to both PS and Ab29 when compared to WT LILRB2 (Fig. 7c-d). Taken together, these results showed that oAβ, PS and Ab29 binding to LILRB2 require both D1 and D2 domains.

After confirming that D1 and D2 are both required for LILRB2 ligand binding, we next sought to identify the key amino acid residues responsible for ligands and Ab29 binding. Considering the high homology between LILRB1 and LILRB2 (85%) which is the highest among all LILRB and LILRA family receptors (Fig. 7e and Supplementary Table 5), single-amino acid mutations were designed to replace non-conserved residues in LILRB2 with the corresponding residues in LILRB1. After consulting the alignment with LILRB1 and the crystal structure of LILRB2, 12 representative single-amino acid mutations were selected at two structurally distinct regions: 1) interdomain interface (D1 and D2): D36G, S48R, L109S, V114E, S193W, H194Y, and S209L; and 2) the distant tip region (N′ of LILRB2) based on reported major structural differences between LILRB1 and LILRB2 [56]: R74P, P75Q, N80K, R105G, and W106R (Fig. 7f-g). The expression levels of all 12 mutants were verified to be similar to WT LILRB2 using anti-HA antibody (Supplementary Fig. 6a). Among the 12 mutated residues, D36G, L109S, and V114E showed a significant negative impact on the binding between PS and LILRB2 over the WT LILRB2 (Fig. 7c). Beyond PS-LILRB2 interactions, the oAβ or Ab29 binding to LILRB2 was also significantly impacted by mutations at amino acid residues D36, L109, and V114E (Fig. 7b&d). The remaining nine mutants showed binding signals for ligands oAβ, PS, and Ab29 similar to those of WT LILRB2 (Supplementary Fig. 6b-d).

## Discussion

In addition to confirming oAβ as a shared ligand for LILRB2 and TREM2, we discovered for the first time that PS is another shared ligand between TREM2 and LILRB2 using multiple approaches including ELISA, BLI, NFAT-GFP reporter cells, and antibody blocking assay. PS is exposed to the outer leaflet of the plasma membrane starting from the early stage of apoptosis, which serves as one of the crucial signals for the recognition and phagocytosis of apoptotic cells [39–41]. TREM2 recognizes PS associated with fibrillar Aβ in lipid membranes and PS exposed on the surface of damaged neurons [57–59]. The recognition of PS by TREM2 was proven to be critical for microglia detection of damage-associated lipid patterns associated with neurodegeneration and to sustain microglia responses to Aβ accumulation [38]. A loss-of-function mutation R47H of TREM2 showed impaired recognition of PS [38]. Our study also demonstrates the specificity of LILRB2 to the apoptotic marker PS instead of PC on normal cells, suggesting that LILRB2 is another regulator for phagocytosis. Since disease-associated-microglia (DAM) gene studies currently reported mostly involve AD mouse models, 5XFAD and APPPS1, and mice have no LILRB2, we are not able to confirm whether LILRB2 is one of the DAM genes [60]. However, LILRB2 was recently identified as a risk gene contributing to Late-onset Alzheimer’s disease (LOAD) in a large genome-wide association study involving over 1 million patients’ data [61]. The *p*-value was found to be 1.56 × 10^9^ for LILRB2 which is comparable to CD33 (2.21 × 10^10^), which is has bee well established to negatively regulate AD in mouse models [47, 61]. Using a recently published single-cell RNA-seq database for Alzheimer’s disease, we found that LILRB2 was upregulated in human AD patient microglia by 4.5-fold over healthy control [62]. In future studies, we will quantify the RNA and protein levels in the brain samples of a large number AD patients and healthy donors [47].

We demonstrated that ligand-mediated LILRB2 and TREM2 activation using chimeric NFAT-GFP reporter cells. Using reporter cells has multiple advantages. First, it allows studying receptor-ligand binding in cellular conditions [43, 44] and avoiding potential artificial effects in biochemical assays such as ELISA and BLI. Second, ligands require no labeling thus allowing not only proteins but small molecules in the binding assay. Third, receptors are expressed on the cell surface, avoiding the need to purify proteins and allowing the construction of a large number of mutants and even libraries for high throughput screening. The observed lipid-induced activation of TREM2 in this study is consistent with previously reported TREM2 activation by lipid ligands in TREM2 reporter cells [38, 63, 64].

We validated that both TREM2 and LILRB2 binds strongly to oAβ, which is consistent with previously reports [19, 20, 24]. One of the important functions of microglia is the clearance of toxic oAβ plaque, which alleviates the plaque buildup and pathogenesis of AD [65]. TREM2 was shown to play a role in microglia phagocytosis of the oAβ-lipoprotein complex [42] and apoptotic cells [66]. TREM2 knockout microglia showed decreased uptake of the oAβ-lipoprotein complex [42]. Also, TREM2 expression mediated the phagocytosis of lipoprotein complexes [42]. Moreover, increasing TREM2 function pharmacologically by agonist mAbs improved microglia oAβ phagocytosis [42, 67]. oAβ-LILRB2 binding has been shown to play a role in neurological function [16]. The mouse LILRB2 ortholog, PirB, is expressed on neuronal growth cones and is associated with synapses [16]. oAβ-PirB interactions were reported to regulate synaptic plasticity causing memory deficits in visual cortex of adult mice [16]. Together with TREM2 and cellular prion protein (PrP^C^), LILRB2 was one of the three major receptors showing strong interaction with Aβ oligomers [16, 19, 20]. However, there was no report on whether LILRB2 plays a role in regulating TREM2’s function in clearing toxic oAβ plaque by microglia. In this study, we demonstrated for the first time that the shared ligands, oAβ and PS, mediate co-ligation of LILRB2 and TREM2 by the BiFc assay. The co-ligation was dependent on the binding between LILRB2 with ligands since the LILRB2 mutant with a swapped LILRB1 D1 domain abolished LILRB2/TREM2 co-ligation.

We next showed the co-ligation between LILRB2 and TREM2 triggered by their shared ligands oAβ and PS caused inhibition of TREM2 signaling using the reporter cells co-expressing the two receptors. We further demonstrated that inhibition of TREM2 signaling is dependent on the ITIM signaling of LILRB2 since LILRB2 with mutated ITIM (Y533F, Y562F, and Y592F) showed no inhibition. Mutating the three tyrosine residues to phenylalanine abolishes the phosphorylation of ITIM motifs and thus the recruitment of phosphatase without significantly affecting the physicochemical properties of LILRB2 [68, 69]. Similar cross-linking or co-ligation-based inhibition of ITAM signaling by receptors bearing ITIM motifs was also observed for PD-1 and the T-cell receptor CD3. The immunoreceptor tyrosine-based switch motifs (ITSMs) in PD-1 play crucial roles in mediating the inhibition of CD3 as demonstrated by mutation studies [69]. The inhibitory signals delivered by PS-LILRB2 interactions are also consistent with the fact that PS serves as a global immunosuppressive signal in efferocytosis, infectious disease, and cancer [70].

TREM2 function deficiency in 5XFAD mice showed reduced colocalization with Aβ plaques [38]. The absence of TREM2 signaling decreased microgliosis as indicated by significantly attenuated morphological transformation to reactive microglia [38]. Macrophages with TREM2 loss-of-function variant R47H show reduced TREM2 function as indicated by reduced ligand binding and phagocytosis of Aβ-lipoprotein complex [42]. Deficiency of TREM2 signaling enhanced autophagy in 5XFAD mouse model and AD patients [71]. In addition, TREM2 deficiency curtails anabolic and energetic metabolism demonstrated by ER stress, defective mTOR signaling, reduced ATP levels, and biosynthesis pathways [71]. Moreover, it was reported that microglia expressing a dysfunctional T66M TREM2 variant showed diminished microglia activity and reduced brain glucose metabolism as measured by small-animal positron emission tomography (μPET) [72]. Considering these crucial roles of TREM2 signaling in microglia function, attenuation of the LILRB2-mediated TREM2 signaling inhibition is desired to restore microglia function such as clearance of toxic oAβ plaque during AD development.

Although TREM2 is largely believed to play a positive role in regulating amyloid pathology, tau pathology studies have revealed opposite trend [73, 74]. For example, PS19-TREM2R47H (PS19 mice with a loss-of-function TREM2 mutation R47H) mice had significantly attenuated brain atrophy and synapse loss when compared to PS19-TREM2CV (PS19 mice with wild type TREM2) mice [73]. The reduced tau pathology was attributed to attenuated microglial reactivity and decrease in phagocytosis of postsynaptic elements in PS19-TREM2R47H mice. However, conflicting findings were also reported that TREM2 deficiency leads to accelerated and exacerbated hyperphosphorylation and aggregation of tau in a humanized mouse model of tauopathy [75]. It was also reported that TREM2 slowed AD progression and reduce tau-driven neurodegeneration by restricting the degree to which β-amyloid facilitates the spreading of pathogenic tau, which suggests an interplay among TREM2, tau pathology, and amyloid pathology [76]. Therefore, therapeutic strategies boosting TREM2 functions or microglial activities should be carefully evaluated in disease stages and more thorough studies in AD models in addition to amyloid pathology.

We showed that both the D1 and D2 domains of LILRB2 are required for ligands oAβ or PS binding, which is consistent with the previous report that both D1 and D2 of LILRB2 contribute to oAβ binding [56]. Based on these findings, we focused on the discovery of ligand blocking antibodies that specifically target the D1 and D2 domains of LILRB2. Using the chimeric LILRB2 NFAT-GFP reporter cell assay, we successfully isolated a panel of D1 and D2 targeting mAbs that block oAβ or PS-LILRB2 interaction and signaling. Among the mAbs, Ab29 was identified with sub-nanomolar IC_50_ (0.1-0.3 nM IC_50_ and 1 nM for complete blocking) in inhibiting LILRB2 signaling induced by either oAβ or PS. In the BiFc assay, Ab29 was able to block the co-ligation of LILRB2 and TREM2 and showed sub-nanomolar activity in attenuating the inhibitory effect to TREM2 signaling mediated by LILRB2. More significantly, the potent activity of Ab29 in attenuating LILRB2-mediated TREM2 signaling inhibition and restoring microglia function such as clearance of toxic oAβ plaque was also demonstrated in vivo. Clearance of soluble oAβ is desired since soluble oAβ works as synaptotoxins leading to cognitive impairment [77, 78]. Clearance of soluble oAβ has always been an important mechanism of anti-Aβ monoclonal antibody therapeutics [79]. Therefore, by promoting oAβ microglial clearance, LILRB2 antagonism serves as a promising approach in AD treatment.

Due to the high sequence similarity of the LILRA and LILRB family of receptors, it is critical for drug discovery purposes that the antibodies target LILRB2 specifically without affecting other receptors. Similarities between LILRB2 and other receptors in the LILRB and LILRA family is from 42% (to LILRB4 ECD) to 80% (to LILRB1 ECD). Among them, LILRB1 shares the highest similarity to LILRB2 with 80% homology. The majority of the antibodies isolated in this study including Ab29 demonstrated high specificity to LILRB2 over other receptors in the LILRB and LILRA families. Cross-reactivity was measured using both Octet and ELISA to ensure coverage of both surface-coated and soluble antigen status.

As one of the important biological functions of TREM2, we studied the effects of Ab29 on TREM2-mediated oAβ-lipoprotein complex phagocytosis. We observed LILRB2-dependent inhibition of oAβ-lipoprotein complex phagocytosis in BV2 expressing LILRB2, human microglia cell line HMC3, and hMGLs. Treatment by Ab29 successfully restored phagocytosis in a TREM2 signaling-dependent manner. When TREM2 signaling was blocked by a TREM2-inhibiting antibody, the effects of the LILRB2 blocking antibody Ab29 were not evident, indicating that the LILRB2-mediated phagocytosis is dependent on the function of TREM2, and LILRB2-mediated oAβ phagocytosis inhibition is upstream of TREM2. A similar relationship was observed for CD33, where mitigation of Aβ pathology by CD33 knockout was abrogated if TREM2 is knockout [61].

The effects of Ab29 on microglia phagocytosis were seen to be consistent with the regulation of TREM2 and LILRB2 signaling pathways. Ab29 treatment significantly increased SYK phosphorylation and decreased SHP1 phosphorylation, indicating the activation and blockade of TREM2 and LILRB2 pathways, respectively. Using hMGLs, we further shown the Ab29 blockade of LILRB2 significantly increased hMGL migration, phagocytosis, and proinflammatory cytokine mRNA levels. Considering the high similarities between hMGLs and human microglia, the improvement in microglia functions by Ab29 is profound and significant.

Our studies on the mechanisms of interactions between LILRB2 with its ligands and Ab29 revealed that both D1 and D2 domains are needed for LILRB2-ligand interactions. We observed specific recognition/binding of LILRB2 instead of LILRB1 by ligands, even though these two receptors share high similarity (80%). Using chimeric reporter cells with D1 or D2 of LILRB2 replaced by D1 or D2 of LILRB1, we confirmed that both D1 and D2 domains are necessary for LILRB2 interactions with oAβ and PS. The observation is consistent with the previous report that mutations in either D1 or D2 significantly affect the binding of oAβ to LILRB2 [56]. Based on the computer modeling, oAβ binds in the groove between D1 and D2 domains, contacting both D1 and D2 [56]. To identify key residues responsible for the binding between LILRB2 and oAβ or PS, a series of mutations were introduced into the LILRB2 D1 and D2 domains by replacing the non-conserved amino acid of LILRB2 with the corresponding amino acid of LILRB1. We choose representative amino acid residues within the interdomain interface between D1 and D2, and the distant tip region of D1, which are responsible for the major structural differences between LILRB1 and LILRB2 [37]. We observed that residues D36, L109, and V114 are most critical for LILRB2 interactions with ligand oAβ, PS, and Ab29, suggesting that neutralizing activity of Ab29 is by directly blocking oAβ and PS binding to LILRB2. Future X-ray crystallography studies will further elucidate the detailed interactions between LILRB2, ligands, and Ab29.

We observed the co-localization of LILRB2, TREM2, and IBA1 in human brain tissues of both healthy donors and AD patients. Due to the limited number of samples, extrapolating such observations to a broader scale should take caution. Future studies using a large number of human brain samples need to be carried out to elucidate the LILRB2, TREM2, and IBA1 relationships on a broader scale.

Previously, LILRB2 and PirB were found to negatively regulate neuronal plasticity by their interactions with amyloid oligomers [16]. Therefore, Ab29 may also improve neuronal plasticity by blocking the interactions with oAβ. Moreover, when designing the antibody, abolishing the Fc-mediated effector functions is necessary, which may cause a potential immune attack of neurons by NK cells and macrophages via Fc receptor-mediated mechanisms such as ADCC and ADCP [80, 81]. It was reported that LILRB2 antagonism inhibited receptor-mediated activation of SHP1/2 and enhanced proinflammatory responses [21]. LILRB2 blockade effectively suppressed granulocytic MDSC and Treg infiltration and significantly promoted in vivo antitumor effects of T cell immune checkpoint inhibitors [21]. For patients with both AD and tumors, more studies are warrented on the potential adverse effects associated with the Ab29 treatment. While we have demonstrated enhanced clustering of microglia around oAβ plaques with a prominent increase in microglial amyloid plaque phagocytosis when 5XFAD mice were treated with the LILRB2 antagonist Ab29 antibody, future studies can use LILRB2 KI 5XFAD mice to gain mechanistic insights into amyloid pathology, neuron damage, transcriptional changes, and animal behaviors [55]. Using ILRB2 KI 5XFAD mice provides another advantage that native microglia are studied instead of implanted microglia. Thus, immune responses from the native microglia to the implanted microglia will be avoided.

The current hMGL implantation model could be modified into a long-term human microglia transplantation model in NSG mice expressing human CSF1, IL3, SCF, and GM-CSF [33]. Such a model will establish a more stable implantation that resembles the native microglia status than the short-term implantation.

In addition to the increased phagocytosis of oAβ and clustering between microglia and amyloid plaques, we noticed LILRB2 blockade significantly increased the inflammatory cytokine mRNA levels in microglia upon stimulation by oAβ-lipid. Such an increase in inflammation could be a double-edged sword depending on the stages of AD. In the early stage, microglia activation and appropriate inflammation are beneficial in promoting the clearance of amyloid plaques. However, at late stages of AD, excessive inflammations could be detrimental in escalating neuronal damage [82–84]. This stage-dependent effect of LILRB2 in AD is consistent with the perplexing roles that TREM2 plays in AD [85, 86]. Therefore, LILRB2 blockade or other treatments to boost the functions of microglia should be combined with diagnostic biomarkers to identify the optimal stage of disease to achieve clinical benefits.

Fahrenhold et al. reported that TREM2 is not expressed by neurons [87]. Although it has been reported that PirB (a mouse protein related to LILRB2) is expressed on neurons, there is no direct proof of LILRB2 expression on neurons [16, 56].. However, considering the distant relations between LILRB2 and PirB (less than 50% similarities), the observation of PirB may not be applicable to human LILRB2. Although LILRB2 expression on neurons is beyond the scope of this study, we could validate LILRB2 expression on neurons and elucidate if the blockade of LILRB2-ligand interactions may affect neurons in future studies.

Considering the broad availability of oAβ and PS in human brain tissues, the cluster-mediated inhibition may take place under certain conditions to prevail the competition from ample number of ligands. Therefore, another meaningful future study is to understand the exact conditions or circumstances when the clustering overcomes the interactions from existing ligands. By elucidating the circumstances, more key molecules or processes involved could be discovered and thus advance our understanding of the role of lipids and oAβ in AD.

## Conclusion

This study demonstrated the expression of LILRB2 and clustering with TREM2 in the human microglia. We discovered that PS is a ligand of LILRB2, and LILRB2 and TREM2 share ligands oAβ and PS by multiple approaches which include ELISA, BLI, and NFAT-EGFP reporter cells. The co-ligation of LILRB2 and TREM2 mediated by their shared ligands oAβ and PS induced inhibition of TREM2 signaling in a LILRB2 ITIM motif-dependent manner. We identified a monoclonal antibody Ab29 with high affinity and specificity to LILRB2. Ab29 potently blocked interactions between LILRB2 and its ligands in cell-based functional assays and rescued TREM2 signaling inhibition by LILRB2 co-ligation. Blocking LILRB2 significantly boosted microglial functions, including migration and inflammatory cytokine response. Ab29 induced more phagocytosis of amyloid by microglia both in vitro and in vivo. This study sheds new light on rescuing TREM2 signaling in microglial cells through blocking LILRB2 suppression of TREM2, and reducing oAβ in AD patients by improving microglia phagocytosis functions.

## Supplementary Information

**Additional file 1: Supplementary Figure 1**. Immunofluorescence staining of human brain tissue with astrocyte and neuron markers. a. Immunofluorescence staining of human brain tissue of normal subjects showed co-localization of LILRB2 and TREM2 with microglial marker IBA1 (top row). The bottom row showed no co-localization of LILRB2 and TREM2 with astrocyte marker GFAP. Scale bar = 20 μm. b-c. Binding kinetics profiles between oAβ and LILRB2 or TREM2. In the association stage, protein A sensor-captured LILRB2-Fc (b) or TREM2-Fc (c) protein was incubated with oAβ at indicated concentrations The amount of oAβ bound onto the sensors was presented as wavelength shift in nanometers (nm). The red dotted vertical line marks the transit from the association stage to the dissociation stage, where the sensors were dipped into kinetics buffer without oAβ allowing free dissociation. The binding kinetics parameters were calculated using a 1:1 binding model with global fitting.

**Additional file 2: Supplementary Figure 2**. Establishment of reporter cell lines for studying LILRB2 and TREM2 a. Schematic diagram showing the cell line LILRB2_muITIM-TREM2. b. Schematic diagram showing oAβ or PS activates LILRB2 chimeric reporter cells. The chimeric LILRB2-ITAM signaling triggers the NFAT pathway upon binding and crosslinking by oAβ or PS, leading to induced GFP expression. The Ig-like domains of LILRB2 are depicted as spheres. The ITAM motifs of the chimeric LILRB2 are from helical and intracellular regions of TREM2, which mediates activation functions after associating with DAP12. c-d. Activation of LILRB2-chimeric GFP reporter cells by oAβ and lipids. Plate-coated oAβ (c) or lipids (d) were incubated with LILRB2-chimeric reporter cells, and the percentage of GFP^+^ cells is shown in the y-axis. Data are presented as mean ± SD (*n* = 4 independent experiments). e. Schematic diagram showing oAβ or PS activates TREM2-DAP12 reporter cells. TREM2-DAP12 binding and crosslinking by oAβ or PS trigger the NFAT pathway leading to induced GFP expression. ITAM motifs in the TREM2 figure are from DAP12. f-g. Activation of TREM2 GFP reporter cells by oAβ or lipids. Plate-coated oAβ (f) or lipids (g) were incubated with TREM2-DAP12 reporter cells. The percentage of GFP^+^ cells is shown on the y-axis. Data are presented as mean ± SD (*n* = 4 independent experiments). h. Comparison of monomer oAβ versus oligomer oAβ in the activation of LILRB2 or TREM2 reporter cells. i. Schematic diagram showing the reporter cell line muLILRB2-TREM2. j. Surface expression of TREM2 and LILRB2 on BV2-LILRB2 cells. Indicated antibodies were used to stain surface receptors on BV2-LILRB2 cells after Fc blocking. The antibody was detected by Alexa Fluor 488-streptavidin, and the fluorescent signals (x-axis) were plotted as a histogram with normalized cell percentage on the y-axis. k. MFI of LILRB2-BV2 or LILRB2_muITIM-BV2 cells stained by indicated antibodies (x-axis) as shown in j. Data are presented as mean ± SD (*n* = 3 independent experiments).

**Additional file 3: Supplementary Figure 3**. LILRB2 targeting antibodies showed potent blocking activity, high affinity, and specificity. a-b. Screening of antibodies inhibiting oAβ or PS-induced activation of LILRB2-chimeric reporter. Plate-coated oAβ (a) or PS (b) was incubated with LILRB2-chimeric reporter cells under the presence of unpurified antibody supernatant (1:20 dilution, antibody name is shown in x-axis). The activation of LILRB2-chimeric reporter cells was observed as percentages of GFP^+^ cells. Data are presented as mean ± SD (*n* = 3 independent experiments). c-d. Titration of blocking activities of purified LILRB2 antibodies against oAβ- or PS-LILRB2 interactions. Plate-coated oAβ (c) or PS (d) was incubated with LILRB2-chimeric reporter cells under the presence of increasing concentrations of purified LILRB2 antibodies (antibody names are shown in the figure legend). The activation of LILRB2-chimeric reporter cells was observed as percentages of GFP^+^ cells. Data are presented as mean ± SD (*n* = 3 independent experiments). e-f. Maximum wavelength shifts of the oAβ- (e) or PS (f)-LILRB2 binding curve in association stage presented in g and h, respectively. g-h. Antibodies blocking LILRB2 binding with oAβ or PS as measured by BLI. LILRB2 was loaded onto protein A sensors via binding with sensor-captured LILRB2 antibodies. The LILRB2-loaded sensors were then incubated with biotinylated oAβ (1 μM, g) or PS (1 mM, h). The amount of oAβ (g) or PS (h) bound onto the sensors is presented as wavelength shift in nanometers (nm). i. Binding kinetics profile between Ab29 and LILRB2 as measured by BLI. In the association stage, protein A sensor-captured Ab29 was incubated with LILRB2-His at indicated concentrations for the designated time presented on the x-axis. The amount of LILRB2-His bound onto the sensors is presented as a wavelength shift in nanometers (nm). The red dotted vertical line marks the transit from association stage to dissociation stage, where the sensors were dipped into kinetics buffer without LILRB2-His allowing free dissociation. j. Titration profiles of purified antibodies binding to plate-coated LILRB2 as measured by ELISA. Data are presented as mean ± SD (*n* = 3 independent experiments). k. Binding of purified LILRB2 antibodies to LILRB2 expressed on HEK293T cells. The percentage of positive staining was gated based on control IgG-treated cells. Data are presented as mean ± SD (*n* = 3 independent experiments). l. Cross-reactivity of all LILRB2 blocking antibodies against other LILRB and LILRA family receptors as measured by ELISA. LILRB2-Fc was included as the positive control. Data are presented as mean ± SD (*n* = 3 independent experiments). m. Cross-reactivity of Ab29 to LILRB and LILRA family receptors as measured by BLI. In the association stage, protein A sensor-captured Ab29 was incubated with Fc fusion proteins of LILRB and LILRA family receptors. The amount of Fc fusion bound onto the sensors was presented as wavelength shift in nanometers (nm). The red dotted vertical line marks the transit from association stage to dissociation stage, where the sensors were dipped into kinetics buffer without Fc fusion receptors allowing free dissociation. LILRB2-Fc was included as the positive control. n. Maximum wavelength shifts of the Ab29-LILR binding curve in association stage as presented in m.

**Additional file 4: Supplementary Figure 4**. Screening of LILRB2 antibodies for rescuing LILRB2-mediated TREM2 signaling inhibition. a-b. LILRB2 antibodies rescue oAβ or PS-LILRB2-mediated inhibition of TREM2 signaling. Plate-coated oAβ (a) or PS (b) was incubated with LILRB2/TREM2 reporter cells in the presence of 10 μg/mL purified LILRB2 antibodies. The activation of LILRB2/TREM2 reporter cells was observed as percentage GFP^+^ cells. TREM2 signaling in the treatment groups was normalized based on the percentage of GFP^+^ reporter cells expressing only TREM2 (set to 100%). Data are presented as mean ± SD (*n* = 4 independent experiments). c. Surface expression of TREM2 and LILRB2 on HMC cells. Indicated antibodies were used to stain surface receptors on HMC cells after Fc blocking by human Fc fragment. The antibody was detected by Alexa Fluor 488-streptavidin, and the fluorescent signals (x-axis) were plotted as a histogram with normalized cell percentage on the y-axis. d. MFI of HMC3 cells stained by indicated antibodies (x-axis) as shown in c. Data are presented as mean ± SD (*n* = 4 independent experiments). e. Immunoblot of phosphorylated SHP1 (pSHP1), SHP1 of HMC3 upon incubation with oAβ-lipoprotein complex with indicated treatments for 1 hour. Results are from total input cell lysate with β-actin as the loading control. f-g. oAβ or PS activation of TREM2 GFP reporter cells. Plate-coated oAβ (f) or PS (g) was incubated with reporter cells expressing either human or mouse TREM2, and the percentage of GFP^+^ cells is presented. Data are presented as mean ± SD (*n* = 4 independent experiments). h-i. Blocking of oAβ or PS-TREM2 signaling by a TREM2 antagonist antibody. Plate-coated oAβ (h) or PS (i) was incubated with reporter cells expressing either human or mouse TREM2 in the presence of TREM2 antagonist antibody or control IgG, the percentage of GFP^+^ cells are shown with an antibody treatment. Data are presented as mean ± SD (*n* = 4 independent experiments). j. The LILRB2 antibody clone 42D1 was validated for not blocking either oAβ- or PS-induced LILRB2 signaling. Plate-coated oAβ or PS was incubated with LILRB2-GFP reporter cells in the presence of soluble 42D1 or control IgG, the percentage of GFP^+^ cells is shown with an antibody treatment. Data are presented as mean ± SD (*n* = 4 independent experiments). k. Plate-coated 42D1 activates LILRB2 signals. Plate-coated anti-LILRB2 clone 42D1 or control IgG was incubated with LILRB2-GFP reporter cells. The percentages of GFP^+^ cells are shown. Data are presented at mean ± SD (*n* = 4 independent experiments). l. Antibody 42D1 showed no recuse of LILRB2-mediated TREM2 signaling inhibition in reporter cells. LILRB2-TREM2 2B4 reporter cells were stimulated with either oAβ or PS co-incubated with Ctrl Ig, 42D1, or Ab29. The TREM2 signaling was detected as increased GFP expression. Data are presented at mean ± SD (*n* = 3 independent experiments). m. Validation of negative controls and quenching method used in the hMGL phagocytosis assay. hMGLs were incubated with oAβ-lipid (fluorescence-labeled) under the conditions labeled on the x-axis. The temperatures are incubation temperature. CytoD means co-incubated with CytoD during phagocytosis. TB means trypan blue, which was added after phagocytosis to quench cell surface FAM signals as described in the method section. Data are presented at mean ± SD (*n* = 3 independent experiments).

**Additional file 5: Supplementary Figure 5**. Ab29 increases microglial responses to amyloid plaques in vivo. a. Representative amyloid plaque-microglia co-localization immunofluorescence staining of 5-month-old 5XFAD mice cortex as treated in Fig. 6a. Scale bar = 20 μm. IBA1, microglia marker; 6E10, amyloid plaque marker. b. Quantification of IBA1 area within 30 μm of amyloid plaques in the cortex of mice treated as described in Fig. 6a. *n* = 5 independent mice. c. Representative amyloid plaque-CD68 co-localization immunofluorescence staining of the cortex of 5XFAD mice treated as described in Fig. 6a. CD68, microglia phagocytic marker. Scale bar = 20 μm. d. Quantification of CD68 area within 30 μm of amyloid plaques in the cortex of mice treated as described in Fig. 6a. *n* = 5 independent mice. e. Quantification of Aβ co-localized with CD68 per plaque in the cortex of mice treated as described in Fig. 6a. *n* = 5 independent mice. For all the data presented, bar graphs with error bars represent mean ± SD. For the statistical analysis, *** *P* < 0.001, two-tailed Student t-test.

**Additional file 6: Supplementary Figure 6**. Identification of key domains and amino acid residues of LILRB2 for ligands and antibody binding. a. HA Ab binding to LILRB2 mutants as measured in chimeric reporter cells. Chimeric NFAT-GFP reporter cells expressing individual mutants of LILRB2 (listed in x-axis) were incubated with plate-coated HA antibody 12CA5. The activation of reporter cells is shown as percentages of GFP^+^ cells. b-d. oAβ, PS, or Ab29 binding to LILRB2 mutants as measured on chimeric reporter cells. Chimeric NFAT-GFP reporter cells expressing individual mutants of LILRB2 (listed in x-axis) were incubated with plate-coated oAβ (b), PS (c), or Ab29 (d). The activation of reporter cells is shown as percentages of GFP^+^ cells. Data are presented as mean ± SD (*n* = 4 independent experiments).

**Additional file 7: Supplementary Table 1**. Titration of blocking activities of purified LILRB2 antibodies against oAβ-LILRB2 interactions.

**Additional file 8: Supplementary Table 2**. Titration of blocking activities of purified LILRB2 antibodies against PS-LILRB2 interactions.

**Additional file 9: Supplementary Table 3**. Binding kinetics parameters between LILRB2 antibodies and LILRB2.

**Additional file 10: Supplementary Table 4**. Titration profiles of purified LILRB2 antibodies binding to plate-coated LILRB2 as measured by ELISA.

**Additional file 11: Supplementary Table 5**. Percentage of similarity of ECDs of LILRB and LILRA family of receptors to the ECD of LILRB2.

## Data Availability

The datasets used and/or analyzed during the current study are available from the corresponding author on reasonable request.

## Abbreviations

AD: Alzheimer disease
ATCC: American Type Culture Collection
BBB: Blood-brain barrier
BiFc: Bimolecular fluorescence complementation
BLI: Bio-Layer Interferometry
CytoD: Cytochalasin D
DAP12: DNAX-activation protein 12 or TYRO protein tyrosine kinase-binding protein
DMPC: 1,2-dimyristoyl-sn-glycero-3-phosphocholine
EC_50_: Half-maximal effective concentration
ECD: Extracellular domain
ELISA: Enzyme-linked immunosorbent assay
FRET: Förster resonance energy transfer
HA: Hemagglutinin
HRP: Horseradish peroxidase
IC_50_: Half-maximal inhibitory concentration
IRES: Internal ribosome entry site
ITIM: Immunoreceptor tyrosine-based inhibition motif
ITAM: Immunoreceptor tyrosine-based activation motif
KD: Affinity constant
Kon: Association rate constant
Kdis: Dissociation rate constant
LILRB2: Leukocyte immunoglobulin-like receptor subfamily B member 2
LILRB: Leukocyte immunoglobulin-like receptor subfamily B
LILRA: Leukocyte immunoglobulin-like receptor subfamily A
MAPK: Mitogen-activated protein kinase
M-CSF: Macrophage colony-stimulating factor
NFAT: Nuclear factor of activated T-cells
oAβ: Oligomer amyloid β
PirB: Paired immunoglobulin-like receptor B
PC: Phosphatidylcholine
PLA: Proximity ligation assay
PS: Phosphatidylserine
SHP1: Src homology region 2 domain-containing phosphatase-1
TREM2: Triggering receptor expressed on myeloid cells 2
TMB: 3,3′,5,5′-tetramethylbenzidine
TEA: Triethylamine
