# Supplementary figures and images for: LILRB2-mediated TREM2 signaling inhibition suppresses microglia functions
Source: Mol Neurodegener. 2022 Jun 18;17:44. doi: 10.1186/s13024-022-00550-y (PMC9206387; doi:10.1186/s13024-022-00550-y)

## Supplementary Figure 1

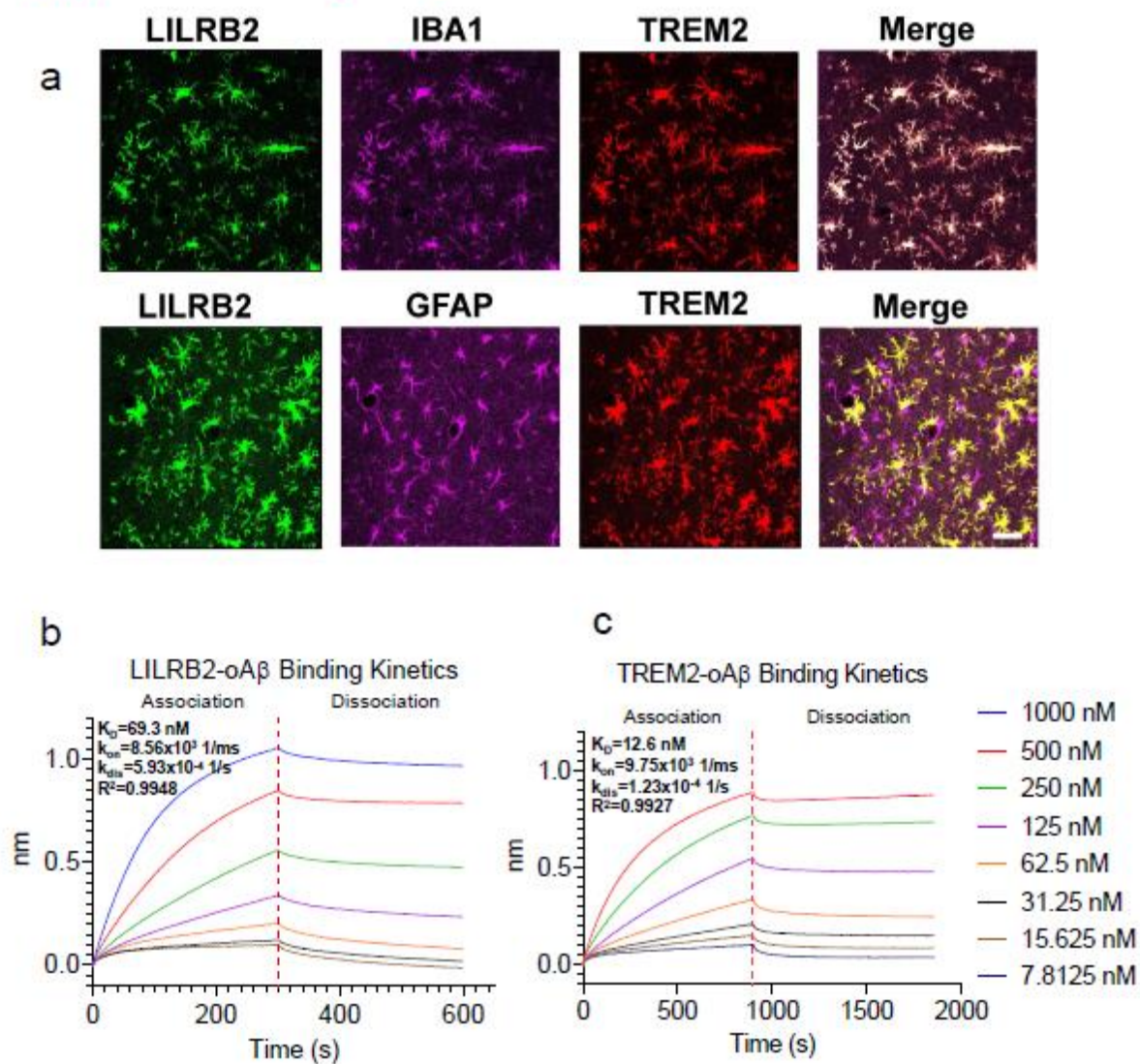

Supplement: Supplementary file 1 — Additional file 1: Supplementary Figure 1. Immunofluorescence staining of human brain tissue with astrocyte and neuron markers. a. Immunofluorescence staining of human brain tissue of normal subjects showed co-localization of LILRB2 and TREM2 with microglial marker IBA1 (top row). The bottom row showed no co-localization of LILRB2 and TREM2 with astrocyte marker GFAP. Scale bar = 20 μm. b-c. Binding kinetics profiles between oAβ and LILRB2 or TREM2. In the association stage, protein A sensor-captured LILRB2-Fc (b) or TREM2-Fc (c) protein was incubated with oAβ at indicated concentrations The amount of oAβ bound onto the sensors was presented as wavelength shift in nanometers (nm). The red dotted vertical line marks the transit from the association stage to the dissociation stage, where the sensors were dipped into kinetics buffer without oAβ allowing free dissociation. The binding kinetics parameters were calculated using a 1:1 binding model with global fitting. [file 13024_2022_550_MOESM1_ESM.pdf]

## Supplementary Figure 2

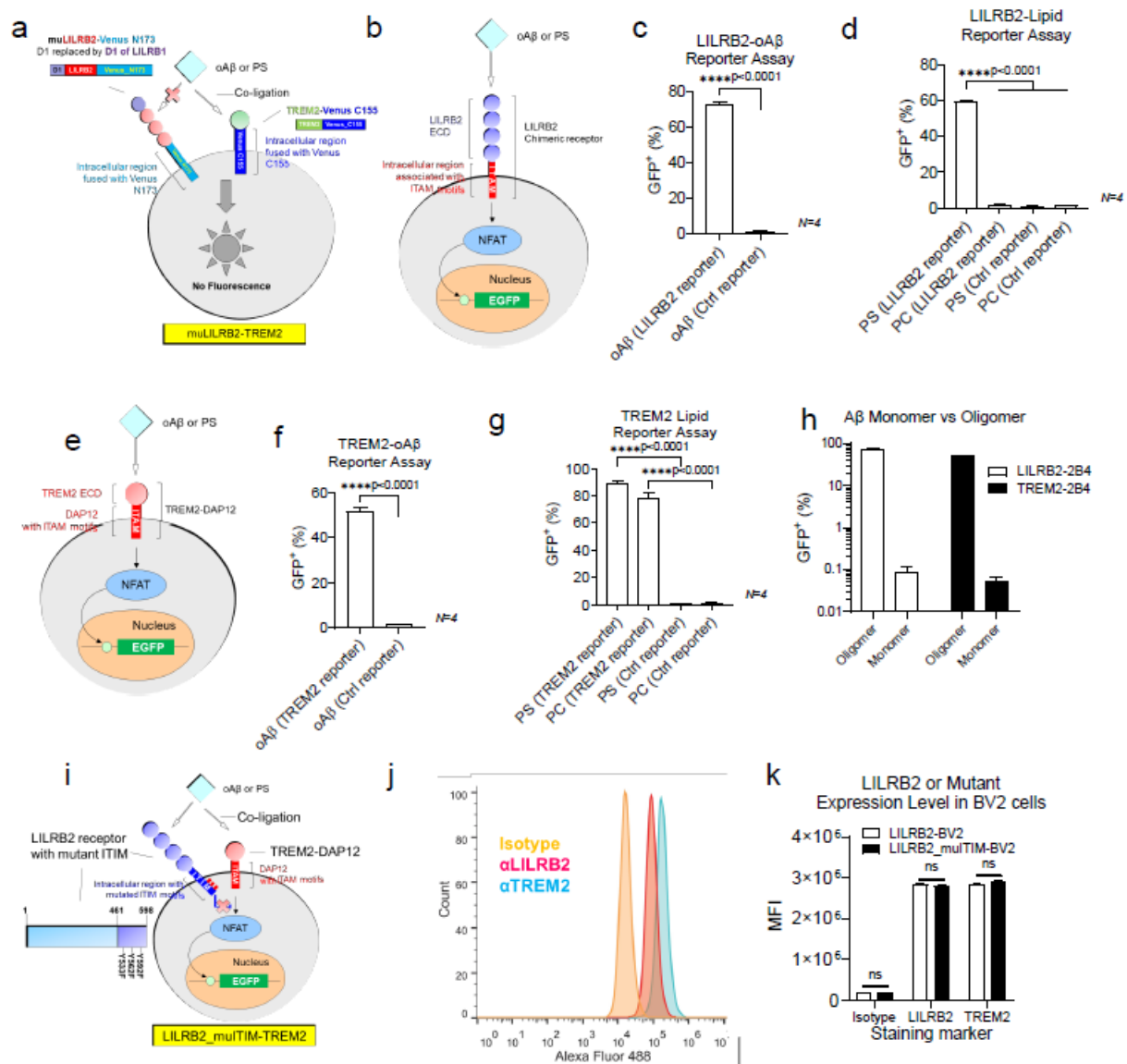

Supplement: Supplementary file 2 — Additional file 2: Supplementary Figure 2. Establishment of reporter cell lines for studying LILRB2 and TREM2 a. Schematic diagram showing the cell line LILRB2_muITIM-TREM2. b. Schematic diagram showing oAβ or PS activates LILRB2 chimeric reporter cells. The chimeric LILRB2-ITAM signaling triggers the NFAT pathway upon binding and crosslinking by oAβ or PS, leading to induced GFP expression. The Ig-like domains of LILRB2 are depicted as spheres. The ITAM motifs of the chimeric LILRB2 are from helical and intracellular regions of TREM2, which mediates activation functions after associating with DAP12. c-d. Activation of LILRB2-chimeric GFP reporter cells by oAβ and lipids. Plate-coated oAβ (c) or lipids (d) were incubated with LILRB2-chimeric reporter cells, and the percentage of GFP+ cells is shown in the y-axis. Data are presented as mean ± SD (n = 4 independent experiments). e. Schematic diagram showing oAβ or PS activates TREM2-DAP12 reporter cells. TREM2-DAP12 binding and crosslinking by oAβ or PS trigger the NFAT pathway leading to induced GFP expression. ITAM motifs in the TREM2 figure are from DAP12. f-g. Activation of TREM2 GFP reporter cells by oAβ or lipids. Plate-coated oAβ (f) or lipids (g) were incubated with TREM2-DAP12 reporter cells. The percentage of GFP+ cells is shown on the y-axis. Data are presented as mean ± SD (n = 4 independent experiments). h. Comparison of monomer oAβ versus oligomer oAβ in the activation of LILRB2 or TREM2 reporter cells. i. Schematic diagram showing the reporter cell line muLILRB2-TREM2. j. Surface expression of TREM2 and LILRB2 on BV2-LILRB2 cells. Indicated antibodies were used to stain surface receptors on BV2-LILRB2 cells after Fc blocking. The antibody was detected by Alexa Fluor 488-streptavidin, and the fluorescent signals (x-axis) were plotted as a histogram with normalized cell percentage on the y-axis. k. MFI of LILRB2-BV2 or LILRB2_muITIM-BV2 cells stained by indicated antibodies (x-axis) as shown in j. Data ar [file 13024_2022_550_MOESM2_ESM.pdf]

## Supplementary Figure 3

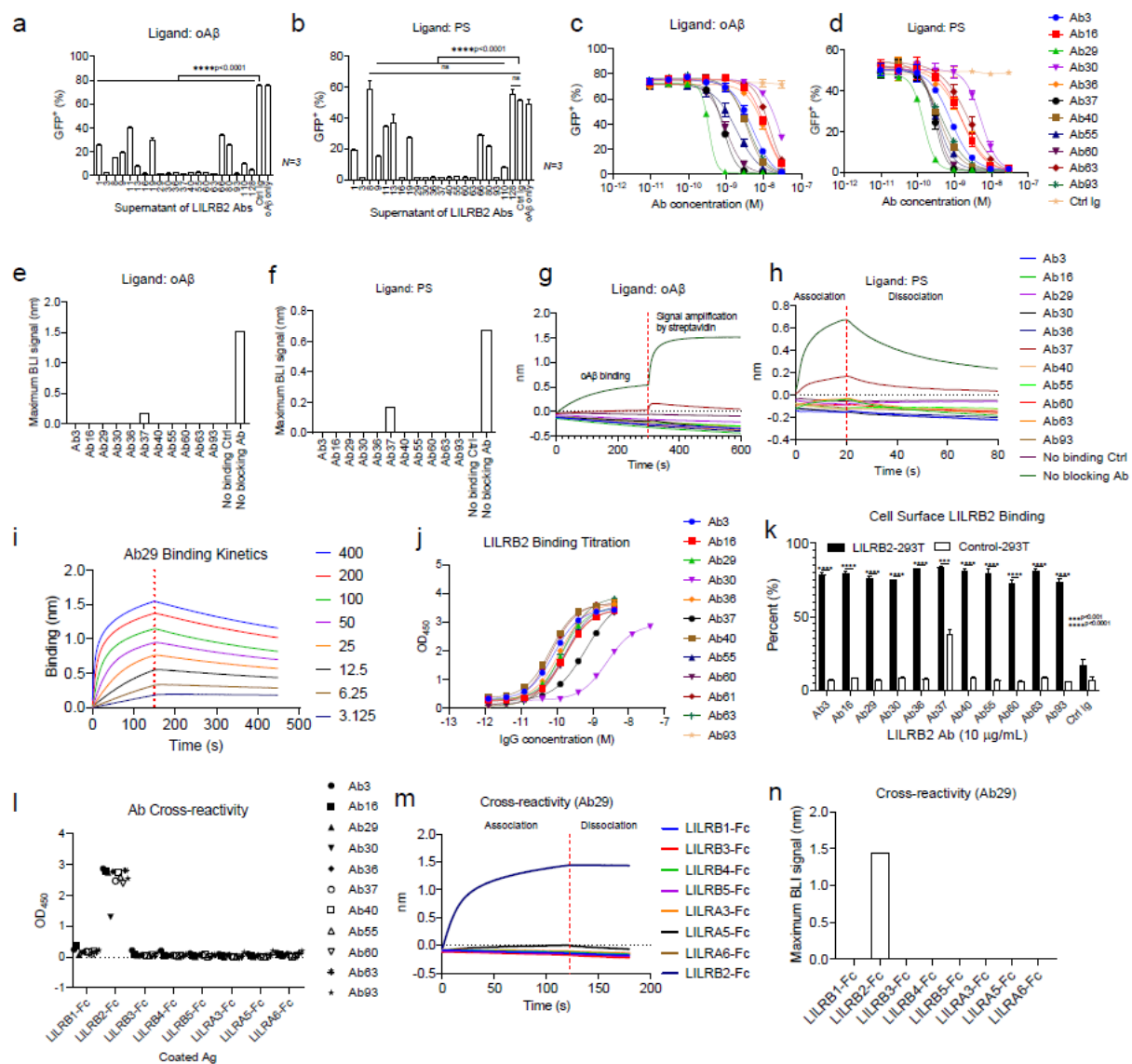

Supplement: Supplementary file 3 — Additional file 3: Supplementary Figure 3. LILRB2 targeting antibodies showed potent blocking activity, high affinity, and specificity. a-b. Screening of antibodies inhibiting oAβ or PS-induced activation of LILRB2-chimeric reporter. Plate-coated oAβ (a) or PS (b) was incubated with LILRB2-chimeric reporter cells under the presence of unpurified antibody supernatant (1:20 dilution, antibody name is shown in x-axis). The activation of LILRB2-chimeric reporter cells was observed as percentages of GFP+ cells. Data are presented as mean ± SD (n = 3 independent experiments). c-d. Titration of blocking activities of purified LILRB2 antibodies against oAβ- or PS-LILRB2 interactions. Plate-coated oAβ (c) or PS (d) was incubated with LILRB2-chimeric reporter cells under the presence of increasing concentrations of purified LILRB2 antibodies (antibody names are shown in the figure legend). The activation of LILRB2-chimeric reporter cells was observed as percentages of GFP+ cells. Data are presented as mean ± SD (n = 3 independent experiments). e-f. Maximum wavelength shifts of the oAβ- (e) or PS (f)-LILRB2 binding curve in association stage presented in g and h, respectively. g-h. Antibodies blocking LILRB2 binding with oAβ or PS as measured by BLI. LILRB2 was loaded onto protein A sensors via binding with sensor-captured LILRB2 antibodies. The LILRB2-loaded sensors were then incubated with biotinylated oAβ (1 μM, g) or PS (1 mM, h). The amount of oAβ (g) or PS (h) bound onto the sensors is presented as wavelength shift in nanometers (nm). i. Binding kinetics profile between Ab29 and LILRB2 as measured by BLI. In the association stage, protein A sensor-captured Ab29 was incubated with LILRB2-His at indicated concentrations for the designated time presented on the x-axis. The amount of LILRB2-His bound onto the sensors is presented as a wavelength shift in nanometers (nm). The red dotted vertical line marks the transit from association stage to dissociation stage, where the se [file 13024_2022_550_MOESM3_ESM.pdf]

Supplementary Figure 4

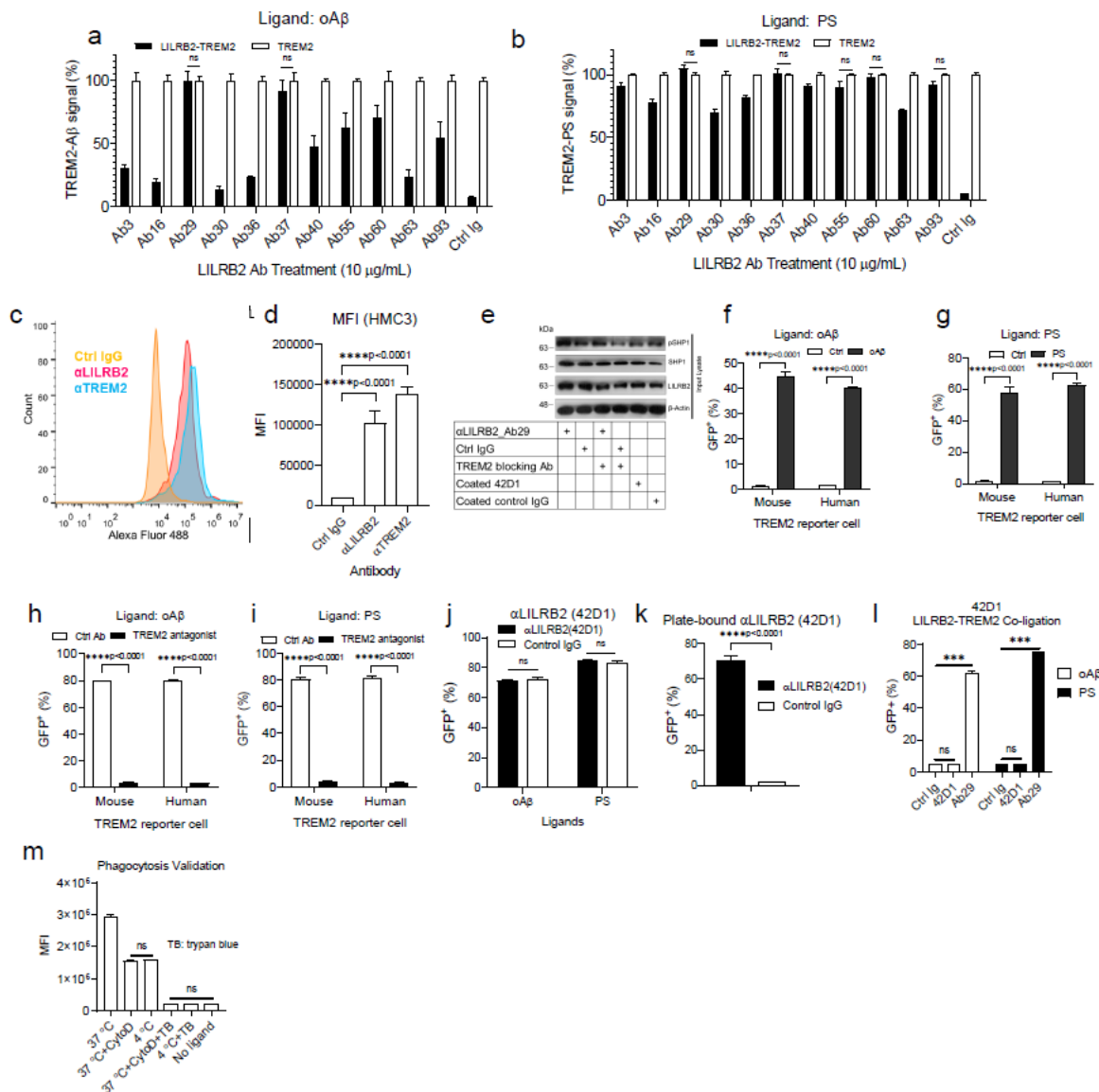

Supplement: Supplementary file 4 — Additional file 4: Supplementary Figure 4. Screening of LILRB2 antibodies for rescuing LILRB2-mediated TREM2 signaling inhibition. a-b. LILRB2 antibodies rescue oAβ or PS-LILRB2-mediated inhibition of TREM2 signaling. Plate-coated oAβ (a) or PS (b) was incubated with LILRB2/TREM2 reporter cells in the presence of 10 μg/mL purified LILRB2 antibodies. The activation of LILRB2/TREM2 reporter cells was observed as percentage GFP+ cells. TREM2 signaling in the treatment groups was normalized based on the percentage of GFP+ reporter cells expressing only TREM2 (set to 100%). Data are presented as mean ± SD (n = 4 independent experiments). c. Surface expression of TREM2 and LILRB2 on HMC cells. Indicated antibodies were used to stain surface receptors on HMC cells after Fc blocking by human Fc fragment. The antibody was detected by Alexa Fluor 488-streptavidin, and the fluorescent signals (x-axis) were plotted as a histogram with normalized cell percentage on the y-axis. d. MFI of HMC3 cells stained by indicated antibodies (x-axis) as shown in c. Data are presented as mean ± SD (n = 4 independent experiments). e. Immunoblot of phosphorylated SHP1 (pSHP1), SHP1 of HMC3 upon incubation with oAβ-lipoprotein complex with indicated treatments for 1 hour. Results are from total input cell lysate with β-actin as the loading control. f-g. oAβ or PS activation of TREM2 GFP reporter cells. Plate-coated oAβ (f) or PS (g) was incubated with reporter cells expressing either human or mouse TREM2, and the percentage of GFP+ cells is presented. Data are presented as mean ± SD (n = 4 independent experiments). h-i. Blocking of oAβ or PS-TREM2 signaling by a TREM2 antagonist antibody. Plate-coated oAβ (h) or PS (i) was incubated with reporter cells expressing either human or mouse TREM2 in the presence of TREM2 antagonist antibody or control IgG, the percentage of GFP+ cells are shown with an antibody treatment. Data are presented as mean ± SD (n = 4 independent experiments). j. The LILRB2 a [file 13024_2022_550_MOESM4_ESM.pdf]

Supplementary Figure 5

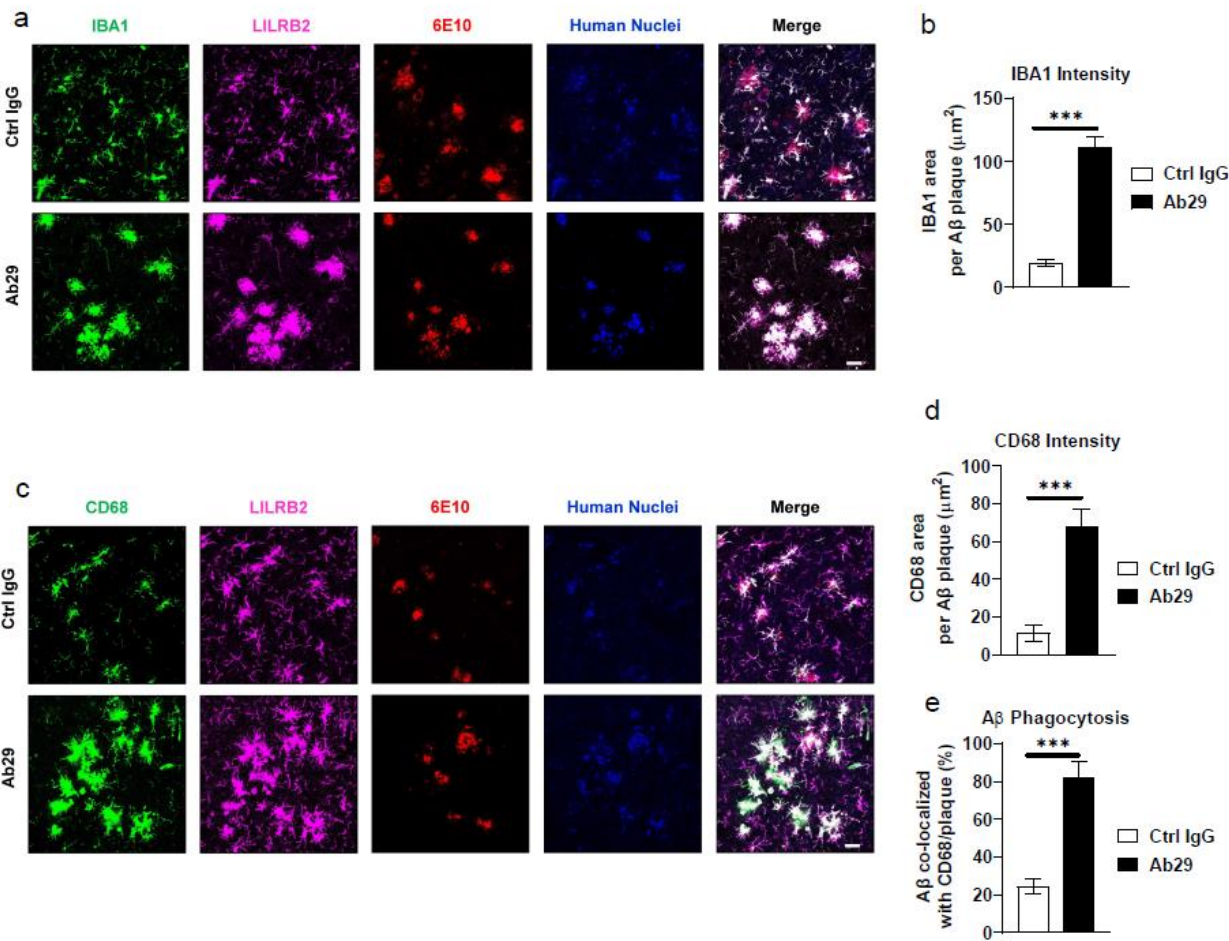

Supplement: Supplementary file 5 — Additional file 5: Supplementary Figure 5. Ab29 increases microglial responses to amyloid plaques in vivo. a. Representative amyloid plaque-microglia co-localization immunofluorescence staining of 5-month-old 5XFAD mice cortex as treated in Fig. 6a. Scale bar = 20 μm. IBA1, microglia marker; 6E10, amyloid plaque marker. b. Quantification of IBA1 area within 30 μm of amyloid plaques in the cortex of mice treated as described in Fig. 6a. n = 5 independent mice. c. Representative amyloid plaque-CD68 co-localization immunofluorescence staining of the cortex of 5XFAD mice treated as described in Fig. 6a. CD68, microglia phagocytic marker. Scale bar = 20 μm. d. Quantification of CD68 area within 30 μm of amyloid plaques in the cortex of mice treated as described in Fig. 6a. n = 5 independent mice. e. Quantification of Aβ co-localized with CD68 per plaque in the cortex of mice treated as described in Fig. 6a. n = 5 independent mice. For all the data presented, bar graphs with error bars represent mean ± SD. For the statistical analysis, *** P < 0.001, two-tailed Student t-test. [file 13024_2022_550_MOESM5_ESM.pdf]

## Supplementary Figure 6

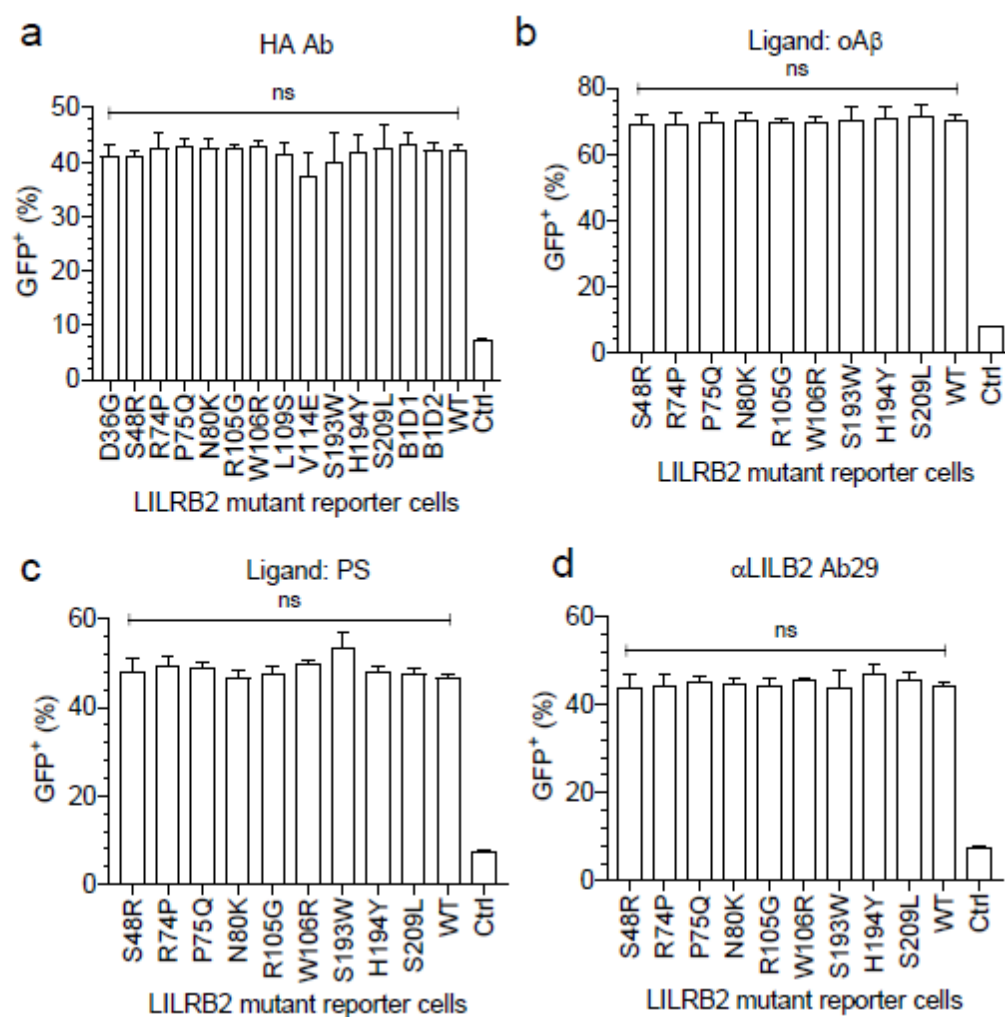

Supplement: Supplementary file 6 — Additional file 6: Supplementary Figure 6. Identification of key domains and amino acid residues of LILRB2 for ligands and antibody binding. a. HA Ab binding to LILRB2 mutants as measured in chimeric reporter cells. Chimeric NFAT-GFP reporter cells expressing individual mutants of LILRB2 (listed in x-axis) were incubated with plate-coated HA antibody 12CA5. The activation of reporter cells is shown as percentages of GFP+ cells. b-d. oAβ, PS, or Ab29 binding to LILRB2 mutants as measured on chimeric reporter cells. Chimeric NFAT-GFP reporter cells expressing individual mutants of LILRB2 (listed in x-axis) were incubated with plate-coated oAβ (b), PS (c), or Ab29 (d). The activation of reporter cells is shown as percentages of GFP+ cells. Data are presented as mean ± SD (n = 4 independent experiments). [file 13024_2022_550_MOESM6_ESM.pdf]
