# Supplementary material for: LILRB2-mediated TREM2 signaling inhibition suppresses microglia functions
Source: Mol Neurodegener. 2022 Jun 18;17:44. doi: 10.1186/s13024-022-00550-y (PMC9206387; doi:10.1186/s13024-022-00550-y)
Supplement: Supplementary file 7 — Additional file 7: Supplementary Table 1. Titration of blocking activities of purified LILRB2 antibodies against oAβ-LILRB2 interactions. [file 13024_2022_550_MOESM7_ESM.docx]

| Ab | IC50 (M) | IC50 95% CI (M) | R squared |
| --- | --- | --- | --- |
| 3 | 5.07E-09 | 4.447e-009 to 5.889e-009 | 0.9944 |
| 16 | 1.1E-08 | 9.590e-009 to 1.402e-008 | 0.9928 |
| 29 | 3.44E-10 | 2.277e-010 to 5.181e-010 | 0.9994 |
| 30 | Not determined | | |
| 36 | 1.13E-08 | 9.400e-009 to 1.685e-008 | 0.9898 |
| 37 | 8.62E-10 | 8.212e-010 to 9.020e-010 | 0.998 |
| 40 | 3.6E-09 | 3.385e-009 to 3.838e-009 | 0.9979 |
| 55 | 1.89E-09 | 1.588e-009 to 2.271e-009 | 0.9896 |
| 60 | 9.82E-10 | 8.943e-010 to 1.079e-009 | 0.995 |
| 63 | Not determined | | |
| 93 | 3.81E-09 | 3.506e-009 to 4.154e-009 | 0.9972 |

Plate-coated oAβ was incubated with LILRB2-chimeric reporter cells under the presence of increasing concentrations of purified LILRB2 antibodies. IC_50_ values were calculated using non-linear curve fitting function log (inhibitor) vs. response -- Variable slope (four parameters) in GraphPad Prism. For antibodies failed to generate a complete titration curve, the IC_50_ values were not calculated and are labeled as not determined.
