# Supplementary material for: LILRB2-mediated TREM2 signaling inhibition suppresses microglia functions
Source: Mol Neurodegener. 2022 Jun 18;17:44. doi: 10.1186/s13024-022-00550-y (PMC9206387; doi:10.1186/s13024-022-00550-y)
Supplement: Supplementary file 8 — Additional file 8: Supplementary Table 2. Titration of blocking activities of purified LILRB2 antibodies against PS-LILRB2 interactions. [file 13024_2022_550_MOESM8_ESM.docx]

| Ab | IC50 (M) | IC50 95% CI (M) | R squared |
| --- | --- | --- | --- |
| 3 | 7.765E-10 | 6.804e-010 to 8.869e-010 | 0.9934 |
| 16 | 1.537E-09 | 1.257e-009 to 1.901e-009 | 0.9859 |
| 29 | 1.415E-10 | 1.345e-010 to 1.490e-010 | 0.9982 |
| 30 | 5.276E-09 | 4.346e-009 to 6.516e-009 | 0.9811 |
| 36 | 1.596E-09 | 1.214e-009 to 2.168e-009 | 0.9796 |
| 37 | 2.929E-10 | 2.663e-010 to 3.190e-010 | 0.9919 |
| 40 | 3.535E-10 | 3.138e-010 to 3.993e-010 | 0.9942 |
| 55 | 3.35E-10 | 3.004e-010 to 3.765e-010 | 0.993 |
| 60 | 3.214E-10 | 3.004e-010 to 3.449e-010 | 0.9957 |
| 63 | 2.392E-09 | 1.932e-009 to 3.074e-009 | 0.9887 |
| 93 | 4.448E-10 | 3.864e-010 to 5.119e-010 | 0.9937 |

Plate-coated PS was incubated with LILRB2-chimeric reporter cells under the presence of increasing concentrations of purified LILRB2 antibodies. IC_50_ values were calculated using non-linear curve fitting function log (inhibitor) vs. response - Variable slope (four parameters) in GraphPad Prism.
