# Supplementary material for: LILRB2-mediated TREM2 signaling inhibition suppresses microglia functions
Source: Mol Neurodegener. 2022 Jun 18;17:44. doi: 10.1186/s13024-022-00550-y (PMC9206387; doi:10.1186/s13024-022-00550-y)
Supplement: Supplementary file 9 — Additional file 9: Supplementary Table 3. Binding kinetics parameters between LILRB2 antibodies and LILRB2. [file 13024_2022_550_MOESM9_ESM.docx]

| Ab | Ab | KD (M) | kon(1/Ms) | kdis(1/s) | Full R^2 |
| --- | --- | --- | --- | --- | --- |
| 3 | Ab3 | 2.72E-08 | 2.08E+05 | 5.65E-03 | 0.9717 |
| 16 | Ab16 | 4.77E-08 | 2.35E+05 | 1.12E-02 | 0.9764 |
| 29 | Ab29 | 9.00E-09 | 1.68E+05 | 1.51E-03 | 0.9879 |
| 30 | Ab30 | 5.49E-07 | 9.21E+04 | 5.06E-02 | 0.9802 |
| 36 | Ab36 | 3.32E-08 | 2.72E+05 | 9.05E-03 | 0.9653 |
| 37 | Ab37 | 8.45E-09 | 4.74E+04 | 4.00E-04 | 0.9967 |
| 40 | Ab40 | 1.92E-08 | 2.45E+05 | 4.71E-03 | 0.9681 |
| 55 | Ab55 | 5.60E-08 | 1.29E+05 | 7.23E-03 | 0.9939 |
| 60 | Ab60 | 2.41E-08 | 2.12E+05 | 5.12E-03 | 0.9856 |
| 63 | Ab63 | 3.67E-08 | 3.10E+05 | 1.14E-02 | 0.9659 |
| 93 | Ab93 | 8.39E-08 | 1.23E+05 | 1.03E-02 | 0.9952 |

The experiment procedures were described in the caption of Figure 3e. The binding kinetics parameters were calculated using 1:1 binding model with global fitting.
