# Supplementary material for: LILRB2-mediated TREM2 signaling inhibition suppresses microglia functions
Source: Mol Neurodegener. 2022 Jun 18;17:44. doi: 10.1186/s13024-022-00550-y (PMC9206387; doi:10.1186/s13024-022-00550-y)
Supplement: Supplementary file 10 — Additional file 10: Supplementary Table 4. Titration profiles of purified LILRB2 antibodies binding to plate-coated LILRB2 as measured by ELISA. [file 13024_2022_550_MOESM10_ESM.docx]

| Ab | EC50 (M) | EC50 95% CI (M) | R^2^ |
| --- | --- | --- | --- |
| 3 | 8.26E-11 | 7.731e-011 to 8.826e-011 | 0.9987 |
| 16 | 1.748E-10 | 1.590e-010 to 1.927e-010 | 0.9976 |
| 29 | 1.374E-10 | 1.301e-010 to 1.453e-010 | 0.999 |
| 30 | 2.494E-09 | 2.272e-009 to 2.752e-009 | 0.9979 |
| 36 | 1.232E-10 | 1.144e-010 to 1.329e-010 | 0.9984 |
| 37 | 6.585E-10 | 5.971e-010 to 7.365e-010 | 0.9991 |
| 40 | 6.645E-11 | 6.222e-011 to 7.096e-011 | 0.9987 |
| 55 | 1.753E-10 | 1.643e-010 to 1.873e-010 | 0.9988 |
| 60 | 1.61E-10 | 1.493e-010 to 1.738e-010 | 0.9983 |
| 63 | 1.445E-10 | 1.366e-010 to 1.531e-010 | 0.9992 |
| 93 | 1.589E-10 | 1.529e-010 to 1.652e-010 | 0.9996 |

Increasing concentrations of purified LILRB2 antibodies were incubated with plate-coated LILRB2. The bound antibody was detected by anti-human F(ab)2 HRP, and the signals were quantified as OD_450_ values. EC_50_ values were calculated using non-linear curve fitting function Sigmoidal dose-response (variable slope) in GraphPad Prism.
