# Supplementary material for: LILRB2-mediated TREM2 signaling inhibition suppresses microglia functions
Source: Mol Neurodegener. 2022 Jun 18;17:44. doi: 10.1186/s13024-022-00550-y (PMC9206387; doi:10.1186/s13024-022-00550-y)
Supplement: Supplementary file 11 — Additional file 11: Supplementary Table 5. Percentage of similarity of ECDs of LILRB and LILRA family of receptors to the ECD of LILRB2. [file 13024_2022_550_MOESM11_ESM.docx]

| Receptor | Similarity (%) |
| --- | --- |
| LILRB1_ECD | 80 |
| LILRB3_ECD | 68 |
| LILRB4_ECD | 42 |
| LILRB5_ECD | 63 |
| LILRA1_ECD | 79 |
| LILRA2_ECD | 76 |
| LILRA3_ECD | 79 |
| LILRA4_ECD | 63 |
| LILRA5_ECD | 49 |
| LILRA6_ECD | 67 |
| PIR-B_ECD | 50 |
